# Förster Resonance Energy Transfer in Luminescent Solar Concentrators

**DOI:** 10.1002/advs.202201160

**Published:** 2022-06-09

**Authors:** Bolong Zhang, Guanpeng Lyu, Elaine A. Kelly, Rachel C. Evans

**Affiliations:** ^1^ Department of Materials Science and Metallurgy University of Cambridge 27 Charles Babbage Road Cambridge CB3 0FS UK; ^2^ CAS Key Laboratory of Design and Assembly of Functional Nanostructures, and Fujian Provincial Key Laboratory of Nanomaterials, Fujian Institute of Research on the Structure of Materials Chinese Academy of Sciences Fuzhou Fujian 350002 China

**Keywords:** Förster resonance energy transfer, light harvesting, luminescent solar concentrator, lumophore, solar energy

## Abstract

Luminescent solar concentrators (LSCs) are an emerging technology to collect and channel light from a large absorption area into a smaller one. They are a complementary technology for traditional solar photovoltaics (PV), particularly suitable for application in urban or indoor environments where their custom colors and form factors, and performance under diffuse light conditions may be advantageous. Förster resonance energy transfer (FRET) has emerged as a valuable approach to overcome some of the intrinsic limitations of conventional single lumophore LSCs, such as reabsorption or reduced quantum efficiency. This review outlines the potential of FRET to boost LSC performance, using highlights from the literature to illustrate the key criteria that must be considered when designing an FRET‐LSC, including both the photophysical requirements of the FRET lumophores and their interaction with the host material. Based on these criteria, a list of design guidelines intended to aid researchers when they approach the design of a new FRET‐LSC system is presented. By highlighting the unanswered questions in this field, the authors aim to demonstrate the potential of FRET‐LSCs for both conventional solar‐harvesting and emerging LSC‐inspired technologies and hope to encourage participation from a diverse researcher base to address this exciting challenge.

## Graphical abstract

Förster resonance energy transfer (FRET) may overcome some intrinsic loss pathways in luminescent solar concentrators (LSCs). In this review, key considerations for the design of efficient FRET‐LSCs from the standpoints of the luminophores and host are outlined. Remaining research questions are highlighted and practical guidelines for FRET‐LSC design are provided to accelerate inclusion and growth in this exciting field.

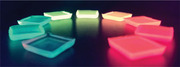

## 1 Introduction

**Figure 1:**
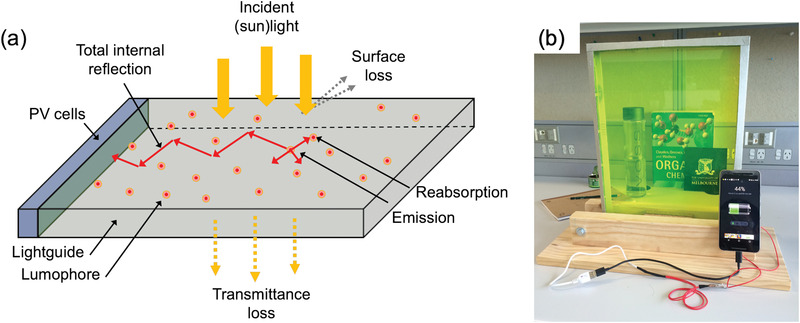
Working mechanism and prototype of an LSC application. a) A typical LSC consists of a lightguide slab that is doped or coated with lumophores. The lumophores absorb incident light and re‐emit it as photoluminescence. The emitted photons are transported by total internal reflectance to the slab edges where they can be collected by attached photovolatic cells. The LSC efficiency is decreased by losses such as transmittance (non‐absorption of incident light), reflectance of incident or emitted light from internal/external surfaces, and reabsorption of emitted photons by neighboring lumophores in the emission path. b) Photograph of a prototype LSC smart‐window (≈30 × 42 cm^2^) charging a cell phone.

The production of renewable energy, particularly through solar photovoltaic technology, has increased dramatically in recent years.^[^ ^1^ ^]^ Traditional PV panels are widely used in open spaces for solar harvesting, but their application in urban or indoor settings is limited due to their bulky and rigid structures and low efficiency under diffuse and low‐intensity light.^[^ ^2^ ^]^ Luminescent solar concentrators are an emerging light‐harvesting technology that complement traditional PV panels, allowing light‐harvesting in atypical environments. A standard LSC consists of a flat lightguide plate, usually made of glass or plastic, which is doped or coated with a luminescent species, or lumophore (**Figure** **1**).^[^ ^3^ ^]^ Incident light is absorbed by the lumophores and emitted at longer wavelengths through photoluminescence. A large portion of the emitted light is concentrated via total internal reflection to the edges of the LSC, where a strip of PV cells may be attached to collect the emitted light and convert it to electrical current.^[^ ^4^ ^]^ Since the basic concept of LSCs was reported in 1976 by Lambe et al.,^[^ ^5^ ^]^ many comprehensive review articles have been published, which help to contextualize the broader research landscape background in this field.^[^ ^6^ ^]^

LSCs are particularly advantageous for use in environments of low‐intensity or diffuse light, such as urban areas, regions of high cloud coverage or indoors.^[^ ^2^ ^]^ They also offer advantages for manufacturing, installation, and design, including lightweight and low‐cost form factors, as well as customizable colors and designs.^[^ ^7^ ^]^ In recent years, new applications for LSCs that exploit these features have been reported,^[^ ^8^ ^]^ for example in urban design as smart windows,^[^ ^9^ ^]^ noise barriers,^[^ ^10^ ^]^ bus stops,^[^ ^11^ ^]^ and greenhouses,^[^ ^12^ ^]^ as well as new technologies such as photomicroreactors,^[^ ^13^ ^]^ wearable electronics^[^ ^14^ ^]^ and photonic temperature sensors.^[^ ^9b^ ^]^

Despite the significant potential, to date commercialization of LSC technology has been limited by its perceived underperformance. While the basic LSC architecture is quite simple at the macroscopic level, there are several competing optical pathways, such as the transmittance of incident light, the escape of photons at the surface due to inefficient trapping, and reabsorption of emitted photons, which lead to energy loss and reduced efficiency (Figure 1a).^[^ ^15^ ^]^ In recent years, there have been an increasing number of reports that demonstrate that some of these losses can be improved through the design of LSCs that exploit Förster resonance energy transfer, often leading to improved device performance.^[^ ^16^ ^]^ FRET is photophysical process in which the excess energy of an excited species, the donor (D), is transferred through space to an emitter (E) species, mediated by dipole–dipole coupling.^[^ ^16a^ ^]^ In contrast to conventional designs, FRET‐LSCs incorporate two or more D–A pairs as lumophores in the photoactive layer.

In this review, we provide a comprehensive overview of FRET‐LSCs that have been developed over the last two decades and discuss the factors responsible for improved device performance, with the specific aim of ensuring the text is accessible to newcomers to this topic. We begin by considering the different energy loss pathways in conventional LSCs and outline the key metrics used to describe LSC performance. We then examine the fundamental principles of FRET and discuss how FRET can be used to overcome the key loss pathways in LSCs. Using key examples from the literature, we will show how the FRET efficiency in LSCs can be enhanced by considering key design criteria, namely the spectral overlap, the photoluminescence quantum yield (PLQY) and the intermolecular distance between the donor and emitter, and the interaction with the host materials. We identify a set of clear guidelines to follow when initiating the bottom–up design of an efficient FRET‐LSC, with the goal of removing barriers to entry to this field. Finally, we highlight current gaps in knowledge in the field and opportunities for further exploration.

## 2 LSC Performance

### 2.1 Energy Loss Pathways

To understand how FRET can boost the performance, we must first consider the four primary energy loss pathways for a typical LSC: i) transmittance; ii) non‐unity photoluminescence quantum yield; iii) escape‐cone losses and iv) reabsorption (Figure 1a).^[^ ^17^ ^]^ The transmittance loss refers to the fraction of incident light that passes through the device without absorption by the lumophore(s). It can be decreased by optimizing the absorption coefficient of the LSC, typically by varying the lumophore concentration.^[^ ^18^ ^]^ The absorption profile of the lightguide material should typically be negligible in the UV–visible region to avoid parasitic absorption losses; however, there are some examples of photoactive lightguides that can act as energy transfer donors in FRET‐LSCs.^[^ ^15c^ ^]^ The PLQY is a measure of how efficiently a lumophore converts absorbed light into re‐emitted photons. In practice, the PLQY is <100%, and is strongly dependent on the intrinsic photophysical properties and concentration of the lumophore(s).^[^ ^19^ ^]^ In general, increased concentration will lead to more unfavorable intermolecular interactions, switching on non‐radiative relaxation channels and thus a decreased PLQY. As such, there is often a trade‐off to be made between minimizing transmittance losses (i.e., increased lumophore concentration) without significant detriment to the PLQY.

The escape‐cone loss denotes the fraction of emitted photons that are not internally reflected towards the LSC edge and instead escape from the top and bottom surfaces of the lightguide.^[^ ^18^ ^]^ It is highly dependent on the refractive index of the lightguide and the orientation of the lumophores.^[^ ^20^ ^]^ The minimum theoretical escape cone loss is 25.9% for typical LSCs made with glass or polymer lightguides with a refractive index of 1.49.^[^ ^20a^ ^]^ However, in practice, escape‐cone losses can be much higher due to scattering from lightguide defects and reabsorption.^[^ ^16c^ ^]^ The final loss pathway, reabsorption, is a phenomenon whereby an emitted photon is subsequently absorbed by an adjacent lumophore in the lightguide matrix.^[^ ^21^ ^]^ A high reabsorption rate can exponentially increase the energy loss due to the escape‐cone and PLQY losses.^[^ ^22^ ^]^ As such, reabsorption can be a major source of inefficiency in LSCs. The Stokes shift (i.e., the separation between positions of the band maxima of the absorption and emission spectra of the same electronic transition) is commonly used as a rudimentary assessment of the potential for reabsorption.^[^ ^23^ ^]^ However, the reabsorption contribution can be calculated more accurately from the spectral overlap integral (i.e., the region of overlap of the absorption and emission spectra) in a single lumophore system.^[^ ^24^ ^]^ In addition, the reabsorption rate has been estimated using computational simulations of LSCs, which yield the average number of reabsorption events per emitted photon during its life cycle (*n*_re − abs_) in the lightguide.^[^ ^25^ ^]^ The value of *n*_re − abs_ can be greater than 1 as each of the primary emitted photons can be reabsorbed and emitted several times during its life cycle. As such, even a small spectral overlap integral can lead to significant reabsorption in the LSC, which increases with the scale of the device.

### 2.2 Performance Metrics

The described energy loss pathways all contribute to the final performance of the LSC. Historically, the discussion and comparison of LSC performance has been challenging due to the absence of clearly defined—and widely accepted—protocols for their characterization. However, great progress has been made in this regard over the last few years and consensus for the measurement and reporting of figures‐of‐merit for bare LSCs^[^ ^26^ ^]^ and integrated LSC‐PV^[^ ^27^ ^]^ is emerging. For clarity, the terminology we will adopt in this review will now be defined.

The optical performance of an LSC is described by the internal photon efficiency, *η*_int_ and the external photon efficie*ncy*, *η*_ext_, defined respectively as

(1) $$\eta_{\mathrm{int}}=\frac{N_{\mathrm{out}}}{N_{\mathrm{abs}}}$$

(2) $$\eta_{\mathrm{ext}}=\frac{N_{\mathrm{out}}}{N_{\mathrm{in}}}$$

where *N*_out_ is the number of photons emitted from the edges of the LSC and *N*_abs_ and *N*_in_ are the number of photons absorbed by, or incident upon, the top surface of the LSC, respectively. Experimentally, *η*_int_ and *η*_ext_ can be measured by irradiating the top surface of the LSC (with all edges uncovered) with solar simulated or monochromatic light (which should be specified) and collecting edge‐emitted photons with an integrating sphere connected to a photodetector.

The geometric gain, *G*, is an important design parameter and represents the theoretical limit of the concentration factor for a given LSC, given by the ratio of the top surface area (*A*_surface_) to the summed area of the edges (*A*_edges_):

(3) $$G=\frac{A_{\mathrm{surface}}}{\sum A_{\mathrm{edges}}}$$

The flux gain, *F*, may be readily derived from the external photon efficiency and the lightguide dimensions, that is,

(4) $$F=G\times \eta_{\mathrm{ext}}$$

We note that *F* is used interchangeably with the concentration factor, *C*, in the literature; we will stick to *F* for consistency in this paper. For *F* > 1, the LSC behaves as a concentrator rather than collector device.

Finally, for integrated LSC‐PV devices, the power conversion efficiency, PCE, is used, given by:

(5) $$\mathrm{PCE}=\frac{I_{\mathrm{SC}}\times \cdot V_{\mathrm{OC}}\times \mathrm{FF}}{P_{\mathrm{in}}\times A_{\mathrm{surface}}}$$

where *I*_SC,_ *V*_OC_, and FF are the short‐circuit current, open‐circuit voltage and fill factor of the PV cell, respectively, and *P*_in_ is the incident power density (W cm^−2^) on the active (top) surface of the LSC. It is important to note that this parameter is highly dependent on the intrinsic performance of the attached PV cell, such that a larger PCE will be obtained when integrating the same LSC with a more efficient PV device.^[^ ^27b^ ^]^

## 3 How Can FRET Mitigate Energy Loss in LSCs?

We have seen that reabsorption losses can result in a significant decrease in the external photon efficiency of an LSC. An effective way to reduce the reabsorption rate is to decrease the spectral overlap between the absorption and emission spectra of the lumophores. Although some lumophores exhibit an intrinsically large Stokes shift, when considering other vital criteria such as PLQY and dispersion in solid‐state host matrices, only a few suitable candidates have emerged for integration with LSCs.^[^ ^28^ ^]^ To overcome this limitation, energy‐transfer systems consisting of multiple lumophores have been increasingly adopted for LSCs.

**Figure 2:**
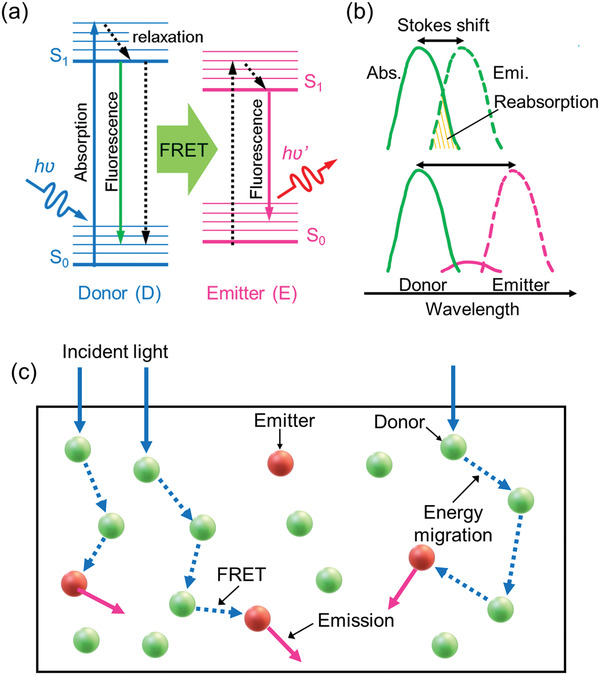
Mechanism of FRET. a) Simplified Jablonski diagram of a typical FRET process following light absorption by the donor. b) FRET helps overcome reabsorption losses, which are common in single lumophore systems exhibiting a small Stokes shift (green—upper). The addition of an emitter (pink) helps reduce reabsorption by increasing the effective Stokes shift. Just a small emitter:donor ratio is required to fully quench the donor's photoluminescence, and the emitter becomes the primary emissive species. c) Schematic representation of FRET between donor and emitter molecules in a lightguide host (black rectangle). Donor molecules absorb the incident light and energy may pass between several donor molecules via energy migration prior to FRET to the emitter species.

The concept of integrating FRET systems with LSCs can be traced back to 1977 when Swartz et al. made a prototype LSC by incorporating two lumophores, Rhodamine‐6G and Coumarin 6, in a poly(methyl methacrylate) (PMMA) plate to broaden the absorption band of the LSC.^[^ ^29^ ^]^ In FRET systems, donor lumophores absorb incident light and transfer the energy to a smaller quantity of emitter lumophores (**Figure** **2**a).^[^ ^30^ ^]^ As the concentration of the donor can be much higher than the emitter, the energy may be transferred through several donors before it reaches an emitter, known as the energy migration process.^[^ ^15a^ ^]^ Thus, the overall absorption spectrum of the LSC consists mainly of the donor characteristics, while the emission spectrum arises from the emitter, resulting in an increased effective Stokes shift of the overall spectrum (Figure 2b).^[^ ^15a^ ^]^ Figure 2c shows a cartoon representation of the FRET process in an LSC, where the donor concentration is greater than that of the emitter. As such, excitation (or exciton) energy migration typically occurs between donor centers before eventually reaching an emitter molecule.^[^ ^31^ ^]^ Ideally, the emission of the donor should be fully quenched by the emitters via FRET, otherwise radiative energy transfer may occur, resulting in additional energy losses.^[^ ^22^ ^]^

FRET is a non‐radiative process, where energy is transferred from the donor to emitter via long‐range dipole–dipole interactions (typically 1–10 nm).^[^ ^16a^ ^]^ The FRET efficiency (*E*) is given by:

(6) $$E=\frac{1}{{\left(\frac{R}{R_{0}}\right)}^{6}+1}$$

where *R* is the average distance between the donor and emitter. The critical FRET radius (*R*_0_, nm) refers to the molecular distance where the FRET efficiency is 50%, defined by:^[^ ^31^ ^]^

(7) $$R_{0}=0.02108\times {\left(\frac{\Phi_{\mathrm{PL}}\kappa^{2}J}{n^{4}}\right)}^{\frac{1}{6}}$$

where Φ_*PL*_ is the PLQY of the donor in the absence of the emitter, *κ* ^2^ is the orientation factor (2/3 for a randomly‐orientated lumophore system), and *n* is the refractive index of the medium. *J* (nm^4^ m ^−1^cm^−1^) is the spectral overlap integral calculated from:

(8) $$J=\int \varepsilon_{A}\left(\lambda \right)F_{D}\left(\lambda \right)\lambda^{4}d\lambda$$

where the absorption spectrum of the emitter is expressed in terms of the molar absorption coefficient (*ε*_A_) per wavelength (*λ*, nm) and the emission spectrum of the donor (*F*_D_) is area‐normalised to unity.^[^ ^32^ ^]^

According to the above equations, the FRET efficiency can be optimized by tuning the spectral overlap integral, PLQY, and the intermolecular distance of the donor and emitters of the lumophore pair system. A sufficient spectral overlap integral requires appropriate alignment of the energy levels of the donor and the emitter (Equation (8)), or in other words strong overlap of the absorption spectrum of the emitter with the emission spectrum of the donor.^[^ ^16a^ ^]^ The FRET efficiency is inversely proportional to the distance between the donor and the emitter to the power of 6 (Equation (6)).^[^ ^33^ ^]^ Therefore, shortening the intermolecular distance, or increasing the localized lumophore concentration, is essential for an efficient FRET system. The FRET efficiency is also directly proportional to the PLQY of the donor (Equation (7)), while the overall PLQY of the FRET system depends largely on the PLQY of the emitters, even if it does not directly affect the FRET efficiency. Accordingly, an ideal FRET lumophore pair will have close‐to‐unity PLQY for both the donor and emitter species.

In the following sections, we will use examples from the recent literature to illustrate how the criteria identified above can be applied to design effective FRET‐LSCs.

### 3.1 Spectral Optimization

The selection of lumophores with the correct spectral characteristics is the first step to designing a new FRET pair for LSCs. Two key criteria must be met: i) there must be sufficient spectral overlap between the donor emission and emitter absorption to ensure high FRET efficiency and ii) for LSC‐PV devices, the total absorption and emission characteristics of the FRET system need to match the solar spectrum and the absorption profile of the attached solar cells.

**Figure 3:**
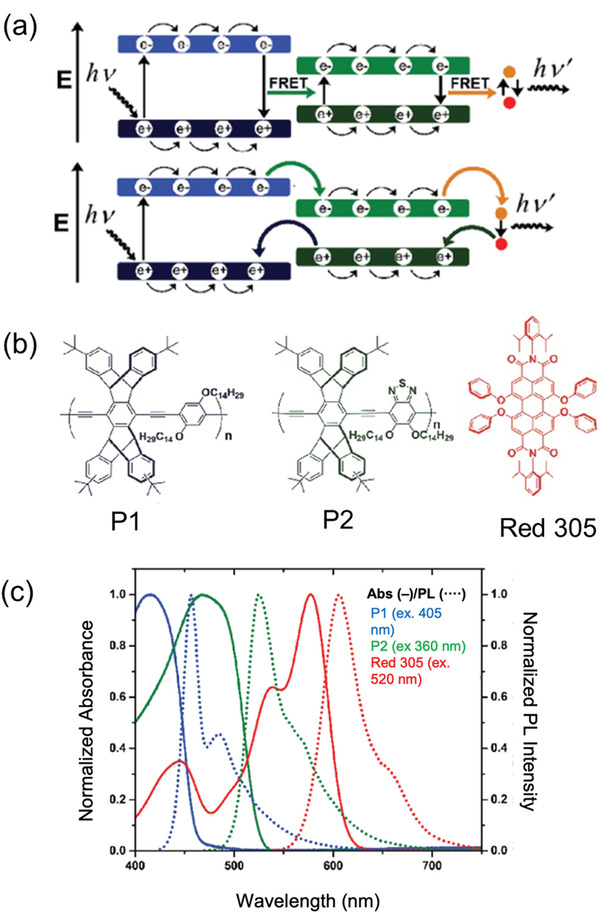
Energy transfer cascades in a tri‐lumophore intermolecular FRET system based on the two conjugated polymer donors (P1, P2) and a molecular emitter (Red 305). a) Proposed energy transfer mechanism. b) Chemical structures and c) absorption and photoluminescence spectra of the donor/emitter species. Adapted with permission.^[^ ^34^ ^]^ Copyright 2016, Wiley‐VCH.

Strong spectral overlap is the easiest requirement to meet, with most reported FRET‐LSCs using lumophore pairs in which the absorption spectrum of the emitter overlaps strongly with the emission spectrum of the donor. However, this is more complicated to achieve in systems containing more than two lumophores, in which one or more species may play the roles of both donor and emitter. A good example of tri‐lumophore intermolecular FRET system was reported by Gutierrez et al. in 2016, which aimed to increase the PLQY and reduce the reabsorption of LSCs.^[^ ^34^ ^]^ The donors were two poly(arylene ethynylene) analogues, named P1 and P2, with a band gap energy of 2.7 and 2.3 eV, respectively, while the widely used molecular lumophore, Lumogen Red F 305 (LR305), was used as the emitter (**Figure** **3**a,b). The emission spectrum of P1 overlaps well with the absorption band of P2 between 430 and 540 nm, while the absorption band of LR305 completely covers the emission of P1, and most of the emission of P2 up to 640 nm (Figure 3c). Bulky tert‐butylated pentiptycene units were added to P1 and P2 to prevent aggregation (and thus non‐radiative relaxation) through *π*–*π* interactions on the polymer backbone. LR305 (1.5 wt%) was directly blended with the two donor polymers at a 1:1 ratio, resulting in an overall PLQY of 79%. Measurements showed that while LR305 made up just 2% of the total absorption of the tri‐lumophore system, the emission spectrum was dominated by LR305, with just a minor emission residue from P2. The authors suggest that both FRET and electron exchange energy transfer (i.e., the Dexter mechanism) occur during the energy transfer cascades in this system (Figure 3a). An LSC was produced by spin‐coating the FRET system on a glass substrate (17.5 × 17.5 × 1.5 mm^3^), and the simulated *η*_ext_ of the device was ≈40% under 405 nm excitation.

The selection of a FRET pair that shows strong overlap with the solar spectrum and an emission output that maps on efficiently with the absorption profile of other light‐harvesting components such as attached solar cells or other lumophore layers is more challenging. A sandwich design was reported by Carlotti et al. in 2016, where an LSC was produced by depositing two lumophore layers separately on both sides of a glass lightguide.^[^ ^35^ ^]^ The top layer contained a single red‐emitting lumophore, either *N,N′*‐bis‐(10‐phenylethyl)‐perylene‐3,4,9,10‐tetracarboxydiimide (PE‐Pery, absorbing from 420 to 600 nm) or LR305 (absorbing from 400 to 620 nm). The bottom layer consisted of a FRET pair of 2,5‐bis(5‐tert‐butyl‐benzoxazol‐2‐yl)thiophene (BTBBT) as the donor, which absorbs strongly between 300 and 420 nm and either PE‐Pery or LR305 as the emitter. The absorption band of the lower FRET layer matched well with the transmission band of the top layer, and by using the same FRET emitter as the lumophore in the top layer, the authors ensured both layers emitted at the same wavelength. A series of LSCs (50 × 50 × 3 mm^3^) were made by casting lumophore layers (25 ± 5 µm) in PMMA (a commonly used lightguide material for LSCs) on the top and bottom surfaces of a glass substrate. The champion device (top: 2 wt% LR305, bottom: 1 wt% BTBBT, 2 wt% LR305) showed an *η*_ext_ of 8.6% and *F* of 1.37. Notably, the sandwich design led to a 14% increase in *η*_ext_ (top: 1 wt% PE‐Pery, bottom: 0.5 wt% BTBBT, 1 wt% PE‐Pery) compared to the analogous single (top) layer LSC, due to the reduced transmittance loss.

Even for FRET pairs showing significant overlap of the donor emission/emitter absorption spectra, the concentration ratio of donor to emitter molecules must also be tailored for LSC use. An unoptimized FRET system shows either high residual donor emission (due to insufficient emitter species within the FRET radius) or excessive absorption from the emitter (due to an excess of emitter molecules), both of which will increase reabsorption and other optical losses.^[^ ^36^ ^]^ Overall, tuning the spectral composition of FRET pairs requires a trial and error approach to some extent, since the concentration ratio of the lumophore(s) may also affect both the PLQY and intermolecular distance. These factors will be discussed in more detail in the following section.

### 3.2 PLQY of FRET Pairs

After selecting a lumophore pair with suitable spectral overlap, the second step in designing an effective FRET‐LSC system usually requires optimization of the PLQY of both the donor and emitter. To achieve a high PLQY, it is necessary to reduce, or even better eliminate, any non‐radiative relaxation channels. In the solid‐state, at the high lumophore concentrations typically needed for efficient FRET, molecular aggregation through intermolecular interactions can lead to aggregation‐caused quenching (ACQ) of the emission.^[^ ^15a^ ^]^ Organic lumophores such as LR305 are commonly used in LSCs, due to their good dispersity in polymeric hosts, high PLQY in solution, low toxicity, and good stability in ambient conditions.^[^ ^15c^ ^]^ However, the planar *π*‐conjugated structure shows a tendency towards *π*–*π* stacking interactions which lead to ACQ.^[^ ^37^ ^]^ Preventing ACQ in organic lumophore based LSCs is therefore a key challenge to overcome to achieve an efficient FRET system.

**Figure 4:**
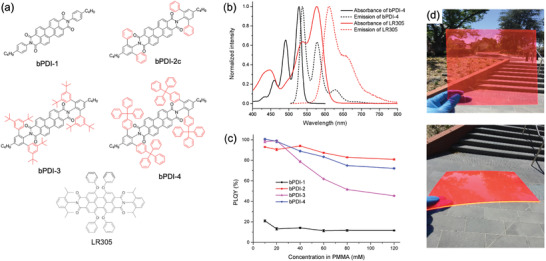
The addition of bulky substituents can inhibit ACQ in perylene diimides. a) Chemical structure, b) absorption and emission spectra, and c) concentration‐dependent PLQY of different perylene diimide structures. d) Photograph of large‐area LSCs (20 × 20 × 0.1 cm^3^) based on the bPDI‐3/LR305 FRET pair. a‐c) Reproduced with permission.^[^ ^19^ ^a]^Copyright 2017, American Chemical Society. d) Reproduced with permission.^[16c]^ Copyright 2019, American Chemical Society.

One approach that has been successfully employed is the design of perylene diimide (PDI) derivatives that contain bulky substituents to reduce *π*–*π* interactions, thereby inhibiting aggregation.^[^ ^38^ ^]^ In 2016, Banal et al. first introduced an FRET pair consisting of a bulky‐substituted PDI (bDPI‐3) as the donor and LR305 as the emitter, embedded in a PMMA substrate (**Figure** **4**a).^[^ ^39^ ^]^ The spectral overlap of the resulting FRET pair was reduced compared to the donor‐only sample due to the 100 nm increase in the effective Stokes shift, while retaining a PLQY of 82% at a high donor loading (58 mm). From the same group, a series of bulky‐substituted PDIs (bPDI‐1 to bPDI‐4, Figure 4a,b) were later designed by Zhang et al. in 2017.^[^ ^19a^ ^]^ The ACQ of the bPDIs was significantly reduced in comparison with bPDI‐1, the unsubstituted counterpart (Figure 4c). In particular, bPDI‐4 showed an emission spectrum consistent with mostly monomer units and a PLQY of 72% at 120 mm in a PMMA matrix, which is high for PDI derivatives at such elevated concentration. On combining bPDI‐4 (60 mm) with LR305 (12 mm) in PMMA as an FRET pair, the donor's emission was fully quenched and the total PLQY increased to ≈90%. Notably, the PLQY of the FRET pair remained >90% when the concentration of bPDI‐4 was increased to 120 mm. A subsequent study extended the bPDI series and further investigated how the photophysical properties were affected by the bulky substituents in high concentration polymeric matrix.^[^ ^19b^ ^]^ A series of Monte‐Carlo ray‐tracing simulations were performed on FRET‐LSCs based on the bPDIs and LR305. The simulated *η*_int_ was 56% for 60 mm bPDI‐4 donor with 12 mm LR305 acceptor at *G* = 120, indicating this FRET pair was an ideal candidate for large‐area LSCs due to the reduced reabsorption.

To demonstrate this potential, Zhang et al. fabricated a large‐area LSC using a bPDI‐3 (60 mm) and LR305 (12 mm) FRET pair (Figure 4d).^[^ ^16c^ ^]^ The lumophores were dispersed in a PMMA matrix and printed as a thin‐film (≈3 µm) on a colorless PMMA sheet (≈1 mm) via doctor‐blade film deposition.^[^ ^40^ ^]^ The PLQY of the FRET pair layer was ≈97% and the spectral overlap was reduced in comparison with the donor alone. The resulting LSC was cut into a square of 20 × 20 cm^2^ and exhibited a PCE of 2.59% under AM 1.5 G illumination, when coupled with perovskite PV cells and a back‐diffuser reflector. At the time of publication, this was the best experimental performance of single‐junction LSC for *G* = 50.

An alternative approach is to accept that aggregation cannot be fully eliminated in the solid state and instead use lumophores that show improved PLQY upon aggregation. In contrast to typical organic lumophores, the emission of aggregation‐induced emission (AIE) active lumophores (i.e., AIEgens) can be enhanced when aggregated in poor solvent or confined in the solid‐state,^[^ ^19^, ^41^ ^]^ due to the deactivation of non‐radiative relaxation pathways via restricted intramolecular rotations.^42^ The sterically‐hindered core structure of AIEgens can also prevent *π*–*π* stacking between AIE molecules at high concentrations in the solid‐state.^[^ ^41^, ^43^ ^]^ Moreover, AIEgens often exhibit large Stokes shifts, high PLQYs, and excellent stabilities,^42^ making them ideal candidates for LSC applications.^[^ ^15^, ^44^ ^]^ The first prototype of an AIE‐based LSC was reported in 2014 when tetraphenylethene (TPE), an archetypal AIEgen, was doped in PMMA film cast onto a glass substrate (*G* = 2.5).^[^ ^44^ ^]^ Even at a high doping concentration of 10 wt%, the device showed no emission quenching. Nevertheless, the emission maximum of TPE ($\lambda_{\max}^{\mathrm{em}}$ ≈ 450 nm) was poorly matched with the band gap of typical silicon solar cells.^[^ ^45^ ^]^ Several subsequent attempts were made to tune the emission of AIEgens by decorating the molecules with electron‐donating or ‐withdrawing moieties.^[^ ^44^, ^46^ ^]^ However, the resulting red‐emitting AIEgens all showed a reduction in their PLQYS when doped in polymer matrices.^[^ ^44^, ^46^ ^]^ In addition, the absorption window of a single AIEgen is often insufficient to cover the whole solar spectrum. An ideal solution is to use FRET to transfer the excitation energy of an AIEgen donor to a red‐emitting emitter with high PLQY, provided that there is sufficient spectral overlap.

The first example of FRET in an AIEgen‐based LSC was reported in 2014 by Banal et al.^[^ ^47^ ^]^ An AIEgen, 2‐(4‐(diphenylamino)phenyl)‐3,3‐diphenylacrylonitrile (DPATPAN), was used as the donor, in conjunction with the red emitter, 4‐(dicyano‐methylene)‐2‐tert‐butyl‐6‐(1,1,7,7‐etramethyljulolidyl‐9‐enyl)‐4H‐pyran (DCJTB). A high concentration (10 wt%) of the donor DPATPAN was mixed with a small amount (0.1 wt%) of the emitter to minimize the reabsorption losses and ACQ of the emitter, while maintaining a high lumophore density for a good FRET efficiency. As expected for an AIEgen, DPATPAN had a near‐unity PLQY of ≈92% at 10 wt% in a PMMA matrix. Due to the high PLQY of the donor and the excellent spectral overlap between DPATPAN and DCJTB, the emission of the donor was fully quenched through the FRET process and transferred to the emitter. Simulations showed that the decrease in the LSC efficiency with increasing *G* was considerably less for a high donor concentration compared to a high emitter concentration, further highlighting the advantages of using an AIEgen as the donor molecule.

**Figure 5:**
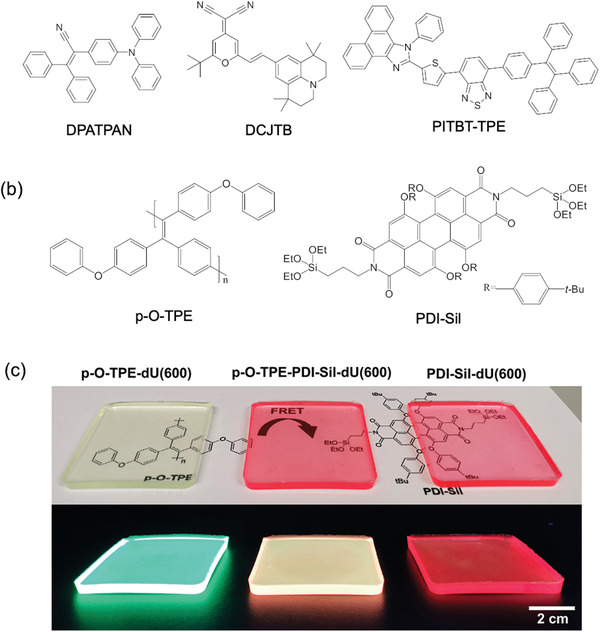
AIEgens can be deployed effectively in FRET‐LSCs. a,b) Chemical structures of reported AIEgens used in FRET‐LSCs. c) Photographs of donor (p‐O‐TPE), donor–emitter (p‐O‐TPE‐PDI‐Sil), and emitter (PDI‐Sil) lumophores in a ureasil (du(600)) lightguide under natural (top) and UV (bottom) light. Adapted with permission.^[^ ^48^ ^]^ Copyright 2019, American Chemical Society.

Subsequent research by the same group in 2018 reported a FRET‐LSC where both the donor (DPATPAN) and emitter (PITBT‐TPE, red‐emitting) were AIE‐active (**Figure** **5**a).^[^ ^25b^ ^]^ Notably, although AIE can reduce the effect of ACQ, the PLQY of DPATPAN still decreases slightly with increasing concentration in the PMMA matrix. This is because non‐radiative relaxation of DPATPAN by intramolecular rotations is intrinsically less important in the rigid environment of the polymer host, which counteracts the positive gains from the AIE effect at high concentrations. Similarly, the PLQY of PITBT‐TPE in PMMA also decreased as a function of concentration, from 93% at 9 mm to 45% at 225 mm. However, upon mixing with DPATPAN (250 mm) in PMMA, the overall PLQY of the FRET pair was higher than for the PITBT‐TPE‐only samples, with full quenching of the DPATPAN emission and a PLQY > 90% observed for a concentration of 22.5 mm of PITBT‐TPE. Monte‐Carlo simulations suggested that the AIE‐FRET enhanced LSCs could give *F* = 11.6 for *G* = 50 under single‐wavelength excitation at 390 nm, which was comparable to other benchmarked LSCs under similar conditions.^[^ ^16b^ ^]^ However, we note that the *F* determined under single‐wavelength excitation is a poor representation of the performance of LSCs in sunlight, particularly at 390 nm where the photon flux is limited under AM 1.5 G (1 sun) conditions.

More recently, Lyu et al. reported a FRET‐LSC that incorporated a polymeric analogue of the TPE AIEgen (p‐O‐TPE, Figure 5b) as the donor in an organic–inorganic ureasil lightguide.^[^ ^48^ ^]^ Polymer lumophores are particularly desirable as energy donors in FRET systems due to the fast exciton migration along their conjugated backbones.^[^ ^34^ ^]^ The p‐O‐TPE donor was mixed with a red‐emitting perylene dye (PDI‐Sil, Figure 5b) in a ureasil lightguide, where the PDI‐Sil emitter was covalently grafted to the host to reduce the extent of its aggregation through a two‐step sol–gel process. Despite the maintained high PLQY at elevated doping concentrations, the formation of large aggregates of p‐O‐TPE gave rise to undesirable scattering losses. Due to effective promotion of AIE in the donor, a moderate PLQY of ≈45% and a large Stokes shift of ≈150 nm were observed. Due to the excellent spectral overlap, FRET was shown to occur from p‐O‐TPE to PDI‐Sil, which increased with acceptor concentration. The optimized LSC (4.5 × 4.5 × 0.3 cm^3^, *G* = 3) with *D*−*A* ratio of 1:1 (by wt%) exhibited an *η*_int_ of 20% under solar simulator excitation, (Figure 5c) which was an improvement on the analogous single lumophore LSCs (*η*_int_ ≈ 9% and ≈18%, for p‐O‐TPE and PDI‐Sil, respectively), demonstrating a viable design utilizing an AIE‐based FRET approach to improve the solar‐harvesting performance.

Despite relatively few reports on the use of AIEgens as lumophores for FRET‐LSCs (and LSCs in general), the results to date suggest this is a promising approach to circumvent detrimental ACQ in FRET systems, providing reduced re‐absorption losses and high PLQY. AIEgen‐based FRET systems that exhibit stimuli‐responsive fluorescence characteristics have also emerged as light harvesters, potentially offering LSCs with additional functionalities.^[^ ^49^ ^]^ The versatile nature of AIEgen chemistry, combined with the growing understanding of their structure–property relationships,^[^ ^19c^ ^]^ will undoubtedly deliver future AIE‐active lumophores with enhanced stabilities, improved PLQY, and tunable emission for the development of highly efficient FRET‐based LSCs.

### 3.3 Shortening the Intermolecular Distance

Once an FRET pair with suitable spectral properties and sufficient PLQY has been chosen, the next step is to apply a strategy that ensures the average intermolecular separation between donor and acceptor molecules falls within the critical FRET radius, *R*_0_. For typical FRET pairs, *R*_0_ is in the range of 2–6 nm.^[^ ^32^ ^]^ Therefore, an effective FRET system should have an emitter concentration > 1 mm, which ensures that about half the population of donors are within 6 nm to the closest emitter.^[^ ^50^ ^]^ In practice, the emitter concentration needs to be greater than 5 mm to sufficiently quench the donor emission. Since the lumophores are often the most expensive component of an LSC slab, such elevated concentrations would make a large contribution to scale‐up costs in the standard bulk/doped LSC architecture. As such, efforts are focused on using bottom–up design strategies to shorten the intermolecular distance through localization of donor–emitter pairs.

#### 3.3.1 Lumophore Localization

**Figure 6:**
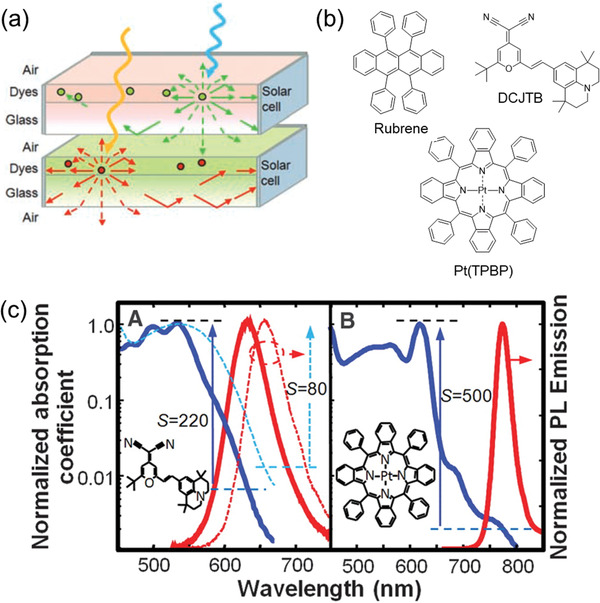
A tandem thin‐film LSC based on FRET layers. a) Architecture of the tandem LSCs, where the top layer absorbs incident light at short wavelengths and the bottom layer absorbs longer wavelengths. b) The chemical structures of rubrene (donor), DCJTB (emitter), and Pt(TPBP) (emitter). c) The normalized absorption and emission spectra of the FRET‐LSC and phosphorescent LSC, respectively. Adapted with permission.^[^ ^16b^ ^]^ Copyright 2008, American Association for the Advancement of Science. The reabsorption factor, *S*, was defined as the ratio of the absorbance at the absorption and emission maxima of the lumophore system.

To improve their efficiency, FRET systems are often designed so that the lumophores are confined in a relatively small space, such as in thin films and particles.^[^ ^16c^ ^]^ The main advantage of this approach is that they are fairly straightforward to prepare. Thin‐film LSCs, in which the donor and emitter are confined in a single thin layer which is cast on a lightguide substrate, are a particularly popular configuration for FRET‐LSCs. In 2008, Currie et al. created a series of tandem thin‐film LSCs (multiple stacked layers of LSCs^[^ ^35^ ^]^), where a fluorescent FRET pair comprised the top layer and either an LSC with a phosphorescent FRET pair or a PV cell was used as the bottom layer (**Figure** **6**a).^[^ ^16b^ ^]^ The fluorescent FRET pair consisted of rubrene and DCJTB as the donor and emitter, respectively (Figure 6b). The phosphorescent FRET pair used DCJTB as the donor and platinum tetraphenyltetrabenzoporphyrin (Pt(TPBP)) as the emitter. Both FRET pairs were blended with tris(8‐hydroxyquinoline) aluminium (Alq_3_), which helped to reduce the aggregation of DCJTB, and was sublimed as a thin‐film on top of a glass substrate (100 × 100 × 1 mm^3^). The authors estimated the degree of reabsorption using the reabsorption factor, *S*, defined as the ratio of the absorbance at the absorption and emission maxima of the lumophore system. The value of *S* was ≈80 when DCJTB was used alone, but increased to 220 when combined with rubrene at a ratio of 30:1, indicating reduced reabsorption in the FRET pair (Figure 6c). It is worth noting that the bottom layer had *S* = 500, due to effective intersystem crossing in Pt(TPBP), leading to emission from a different electronic (triplet) state. The *F* values were 11 and 7 for the resulting LSCs (*G* = 45) based on the fluorescent and phosphorescent FRET pairs, under monochromatic excitation at 534 and 620 nm, respectively. In comparison, the DCJTB‐only LSC had *F* = 9. For the tandem LSCs, the estimated PCE was 6.1% when the fluorescent FRET‐based top LSC (coupled with GaInP PV cells) was paired with the phosphorescent FRET‐based bottom LSC (coupled with GaAs PV cells), and 13.8% when paired with the Cu(In, Ga)Se_2_ PV cells.

Another effective approach is to embed highly‐concentrated lumophores into polymer particles and blend them into the bulk lightguide of the LSC. One such example is a tri‐lumophore FRET system comprising a naphthalene diimide derivative (NDI, absorbing 300–400 nm) and PDI derivative (absorbing 450–550 nm) as primary and secondary donors, respectively, and LR305 as the emitter, reported in 2018 by Pandya et al.^[^ ^51^ ^]^ The emission spectra of NDI and PDI overlap significantly with the absorption spectrum of LR305, meaning both donors can absorb incident light and transfer energy to the emitter via FRET. In addition, the two donors themselves formed a second FRET pair, as the absorption spectrum of PDI overlaps with the emission spectrum of NDI. The overall PLQY of the tri‐lumophore FRET system was ≈95%, much higher than the ≈10% PLQY of the NDI alone, as non‐radiative relaxation of the NDI donor is bypassed. The lumophores were blended at a high concentration (≈32 mm) in PMMA, which was then ground into powder and redistributed in a lightguide made of a lauryl methacrylate (LMA)/ethylene glycol dimethacrylate (EGDM) copolymer. This strategy ensured a high local concentration of the FRET pairs was retained within the bulk‐doped LSC. Monte‐Carlo simulations suggested that an LSC (100 cm^2^) based‐on the optimized tri‐lumophore FRET system could lead to *F* ≈ 12 and ≈8 when coupled with perovskite or Si PV cells, respectively.

**Figure 7:**
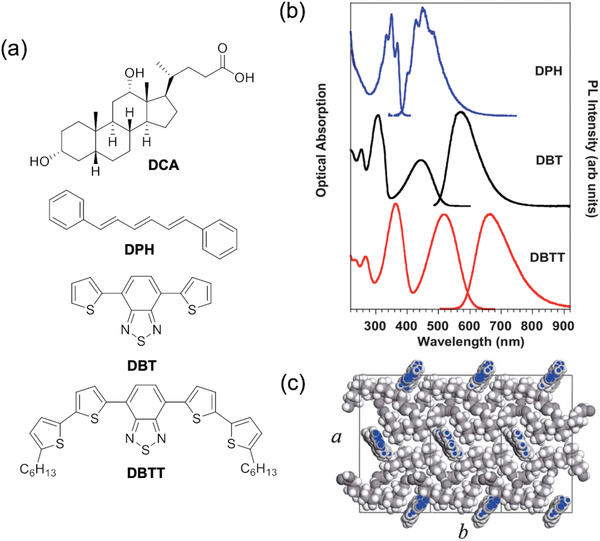
Self‐assembly can be used to localize FRET pairs. a) Chemical structure of the host material DCA and the three lumophores used in the FRET cascade. b) The absorption and emission spectra of the three lumophores. c) Structure of DPH–DCA host–guest system. Adapted with permission.^[^ ^52^ ^]^ Copyright 2013, Royal Society of Chemistry.

Bottom–up self‐assembly approaches are also a promising way to localize FRET pairs. Botta et al. reported a tri‐lumophore FRET system in which the traditional polymer support was replaced by deoxycholic acid (DCA) (**Figure** **7**a), a self‐assembling host matrix containing linear hydrophobic molecular cavities of ≈1 nm diameter.^[^ ^52^ ^]^ Three organic lumophores, diphenylhexatriene (DPH), 4,7‐di(2‐thienyl)‐benzo[2,1,3]thiadiazole (DBT), and 4,7‐bis(50‐hexyl‐2,20‐bithienyl‐5‐yl)2,1,3‐benzothiadiazole (DBTT), whose spectra overlapped to create an FRET cascade (Figure 7b), were blended with DCA and deposited as thin‐film samples (on quartz substrates). As all three lumophores were rod‐shaped, they could be effectively captured by the linear cavities in the DCA matrix (Figure 7c). Notably, the emission spectra of the lumophores within the DCA host were similar to those in dilute solution, indicating that ACQ can be reduced through isolation in these cavities. In the optimized tri‐lumophore system, the emission was dominated by the emitter, with a total PLQY of 67%. Although not discussed by the authors, it is possible that the FRET efficiency in this system is enhanced by the homotropic alignment in the DCA matrix. We note that although this tri‐lumophore FRET system was designed for LSC applications, this work did not report any device fabrication or simulations to demonstrate this.

Another small molecule FRET assembly system based on cocrystals of anthracene (Ac) and tetracene (Tc) was reported in 2014 by Griffini et al.^[^ ^42^ ^]^ LSC samples were prepared by dissolving PMMA, Ac, and Tc in different hot organic solvents and spin‐coating the mixtures as thin films on glass substrates. After film deposition, Ac/Tc cocrystals were observed to form in the PMMA matrix via optical microscopy, facilitating the favored head‐to‐tail transition dipole alignment that occurs upon crystal formation.^[^ ^53^ ^]^ The absorption spectrum of the cocrystals was dominated by Ac and the emission by Tc, with the PL of Ac almost fully quenched. The optimized LSC (25 × 25 × 1.8 mm^3^, *G* ≈ 3.5) was cast using a solution of PMMA:Ac:Tc = 1000:250:1 (by weight) in toluene, resulting in an *η*_ext_ and *F* of 23.72% and 0.83, respectively. In comparison, the *η*_ext_ and *F* of the Ac‐only LSC were only 12.21% and 0.42, respectively. It is worth mentioning that this strategy may not be suitable for other small‐molecule FRET systems, as ACQ can be dominant under crystalline conditions.

A similar approach has been demonstrated for FRET‐LSCs based on perovskite nanoparticles.^[^ ^45^ ^]^ Luminescent materials made from metal halide perovskite nanomaterials are currently drawing significant attention across many fields due to their unique photophysical properties, such as high absorption coefficients, narrow and tunable emission spectrum, high PLQY, and long fluorescence lifetime.^[^ ^54^ ^]^ These properties have naturally sparked interest in the use of perovskite nanomaterials as lumophores in LSCs.^[^ ^55^ ^]^ In an effort to systematically increase the Stokes shift, while maintaining a high PLQY, Wei et al. designed a FRET process based on ensembles of layered perovskite nanoplatelets (PNPLs) for LSC applications.^[^ ^45^ ^]^ Nanoplatelets were made by separating sheets of 3D perovskite nanocrystals with a structure of R_2_(MA)*_n_*_− 1_Pb*_n_* X_3_ *_n_*_+ 1_ (R  =  bulky cation, MA = methylammonium, X  =  Cl/Br/I), where *n* is the number of stacked inorganic sheets in one layer. Due to the quantum confinement effect, the bandgap of the nanoplatelets is dependent on the thickness of each layer, which could be controlled by tuning the ratio between MA and the bulky cations (phenylethylammonium or hexylammonium). PNPLs of different thickness were prepared, in which energy could be transferred from thinner (donor) to thicker (emitter) layers. An ultrafast energy transfer rate (≈0.1 ps^−1^) was observed, indicating the presence of an efficient FRET cascade in the PNPLs. Thin‐film LSCs were prepared by casting a blend of PNPLs and polystyrene on glass substrates (2.5 × 2.5 cm^2^). Films with an optimized *D* *–E* distribution gave an overall PLQY > 75% and *η*_trap_ ≈ 60%. A large‐area LSC (10 × 10 × 0.2 cm^3^, *G* = 12.5) was prepared by rod‐coating a mixture of the optimized PNPLs and PMMA on glass substrates, resulting in an *η*_int_ of 26% for the final device. The *η*_ext_ of the large‐area LSC was only 0.87% mainly due to its high transmittance in the visible range.

#### 3.3.2 Polymers, Dendrimers, and Complexes

**Figure 8:**
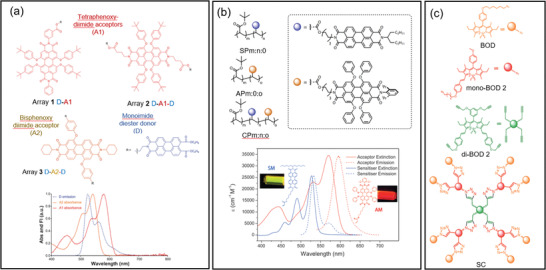
Polymer and dendrimer‐based intramolecular FRET systems. a) Chemical structures of the donor, emitter, and intramolecular FRET trimers reported by Webb et al. The inset figure shows the absorption spectra of the emitters overlap well with the PL spectrum of the donor. Adapted with permission.^[^ ^57^ ^]^ Copyright 2016, Royal Society of Chemistry. b) Chemical structures of the copolymers, and the donor and emitter monomers of the FRET system reported by Davis et al., and the corresponding absorption and emission spectra of the donor and emitter monomers. Adapted with permission.^[^ ^56^ ^]^ Copyright2016, Royal Society of Chemistry. c) Chemical structures of the BODIPY monomers and corresponding FRET dendrimer reported by Bozdemir et al. Adapted with permission.^[^ ^60^ ^]^ Copyright 2011, Wiley‐VCH.

Covalent grafting of *D‐E* pairs onto polymer, dendrimer, or complex structures offers another approach to tune the separation for intramolecular FRET.^[^ ^56^ ^]^ This method offers the additional advantage of reducing ACQ by improving the lumophore dispersity in host matrices.^[^ ^34^ ^]^ In 2016, Webb et al. reported two intramolecular FRET systems consisting of a PDI emitter subunit linked with one or two perylene mono‐imide (PMI) donor subunits (**Figure** **8**a).^[^ ^57^ ^]^ Two different PDI emitter subunits were used, named A1 and A2. The PL spectrum of the donor overlapped well with the absorption spectra of both emitters, resulting in a relatively long *R*_0_ of ≈5.5 nm for both FRET systems (Figure 8a). The FRET efficiency was >99.9% based on the fluorescence lifetime of the donor. These intramolecular FRET trimers (DA1D and DA2D, Figure 8a) were later evaluated for their potential LSC performance using Monte‐Carlo ray‐tracing simulations.^[^ ^58^ ^]^ As expected, the simulated relative *F* (where *F* was normalized to the maximum performance of an emitter‐only sample) of the trimer LSCs outperformed LSCs based on the emitter monomers at low concentration (<2 × 10^−4^ m). However, the *F* of the single emitter LSCs was higher than the trimer LSCs when the concentration increased to ≈3 × 10^−4^ m. According to the authors, this arose due to the red‐edge emission of the trimers being slightly broader than that of the emitters, causing more losses via reabsorption at high concentrations. The donor units also contributed less to the reduction of transmittance losses at high concentrations. The authors suggest that the donor to emitter ratio should be high to minimize reabsorption from the emitter. A very similar intramolecular FRET system was reported by the same research group for a system containing a PDI emitter core covalently linked with four PMI donor units, but it has not yet been used in an LSC platform.^[^ ^59^ ^]^

In 2016, Davis et al. reported a intramolecular FRET system with donor and emitter substituents on the same polymer backbone.^[^ ^56^ ^]^ Two PDI acrylate monomers were incorporated with tert‐butyl acrylate as pendant copolymers (Figure 8b). The polymer backbones provided a scaffold with controlled spacing between the lumophores, which reduced the ACQ of PDIs at high concentrations. The calculated *R*_0_ was 7 nm and the attachment to the polymer chain did not affect the optical properties of the PDI monomers (Figure 8b). Two FRET copolymer films were prepared, with a molar ratio of tert‐butyl acrylate to the donor and the emitter of 150:5:1 and 300:10:1, respectively. While both film compositions had an estimated *D‐E* distance shorter than *R*_0_, the latter had a reabsorption factor *S* = 33.7. In later work, an additional spacer monomer was added to this FRET system by Yasarapudi et al.^[^ ^61^ ^]^ Specifically, the donor monomer was copolymerized with methyl methacrylate, while the emitter monomer was copolymerized with *t*ert‐butyl acrylate. As the *D* unit was highly prone to aggregation, the presence of both intra‐ and intermolecular spacers effectively reduced aggregation and enhanced the PLQY (≈80%) of the FRET system.

Besides linear polymers, dendritic structures also allow lumophore units to be assembled at short distances. The first report of a dendritic FRET system used in LSCs was in 2011 by Bozdemir et al.^[^ ^60^ ^]^ The FRET dendrimer consisted of three different boron‐dipyrromethene (BODIPY) subunits, linked to each other via Huisgen‐type click connections (Figure 8c). The energy levels of the three BODIPY subunits were in a cascade to allow energy to be transferred from the *D* to *E* subunits (Figure 8c), with an overall FRET efficiency > 90%. The *S* factor of the FRET dendrimer was ≈10 000, indicating potential for reduction in the reabsorption effect. Two epoxy resin lightguide samples were made. One was embedded with the FRET dendrimer and the other was produced using a mixture of monomers. Distance‐dependent edge emission measurements and Monte‐Carlo simulations demonstrated increased edge emission at long wavelengths for the FRET dendrimer LSC compared with the monomer‐mixture sample, indicating successful energy transfer.

**Figure 9:**
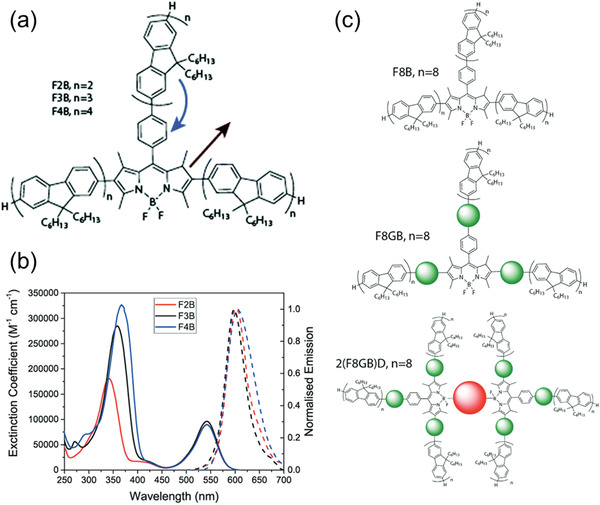
An FRET dendrimer for LSCs. a) Chemical structures of the three FRET dendrimers based‐on a BODIPY core with three oligofluorene side‐chains. b) Absorption and emission spectra of the FRET dendrimers. c) Chemical structures of hypothetical FRET dendrimers structures predicted to show improved LSC performance through Monte‐Carlo simulations. Adapted with permission.^[^ ^62^ ^]^ Copyright 2017, Royal Society of Chemistry.

Davis et al. used a similar strategy to design a series of FRET dendrimers consisting of a BODIPY emitter core linked to three oligofluorene donor side‐chains (**Figure** **9**a).^[^ ^62^ ^]^ The *D:E* ratio was tuned by adding fluorene units to the oligomer chains, which also red‐shifted the donor's emission slightly due to the extended *π*‐conjugation (Figure 9b). The emission of the oligofluorene was fully quenched and the overall PLQY of the three FRET dendrimers was 66–75%. A series of LSCs (10  × 10 × 0.3 cm^3^) were prepared by blending each of the FRET dendrimers in a polymer matrix of LMA:EGDM(4:1 by volume). The best *η*_ext_ among the three LSCs was only 2.44%, mainly due to the narrow absorption bandwidth of the FRET system. Monte‐Carlo simulations were also performed, and the simulated performance of the LSCs aligned with the experimental results. Three further hypothetical structures were also simulated, with an increased number of fluorene units, an intermediate lumophore, which can undergo FRET with both BODIPY and oligofluorene, and a deep‐red emitter introduced into the dendritic structure (Figure 9c). The simulated *η*_ext_ reached 13.4% when the hypothetical FRET dendrimer with the deep‐red emitter was used in the LSC.

**Figure 10:**
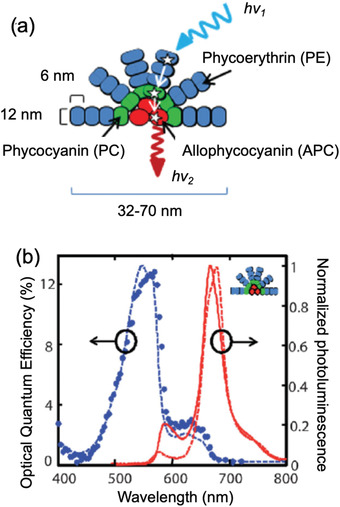
A FRET‐LSC based on a natural light‐harvesting antenna system. a) The structure of the phycobilisome consists of three lumophores self‐assembled in an FRET cascade. b) Absorption and PL spectra of the phycobilisome. Adapted with permission.^[^ ^63^ ^]^ Copyright 2009, Wiley‐VCH.

A natural light‐harvesting FRET‐based antenna system has also been investigated for LSCs by Mulder et al. in 2009.^[^ ^63^ ^]^ Phycobilisome, a water‐soluble pigment‐protein complex found in algae and cyanobacteria, was extracted directly from the organism *Porphyridium cruentum* and bound with three molecular lumophores (phycoerythrin (PE), phycocyanin (PC), and allophycocyanin (APC)). The result was a self‐assembled, cascading FRET pathway. The short‐wavelength PE donors assembled at the outer branches of the complex, and the long‐wavelength APC unit emitters were in the core, while the PC molecules formed the linker between PE and APC (**Figure** **10**a). The ratio of the different lumophores was PE:PC:APC = 408:108:72, and the PLQY of the emitter was 68%. A flexible LSC (22 × 22 × 0.5 mm^3^) was cast by blending the phycobilisome in a polyacrylamide hydrogel. The composition of the hydrogel was optimized to mimic the native environment of the phycobilisome, while retaining enough rigidity as the lightguide. The donor's emission was mostly quenched (Figure 10b), but its PLQY dropped to <50% in the LSC, possibly due to the rigid environment provided by the hydrogel. The final LSC showed a *η*_ext_ of 12.5%, measured using a monochromatic excitation source at 560 nm (matched to the absorption band of PE). An aqueous LSC based on phycobilisome was also made, but no improvement in *η*_ext_ was observed. However, the reabsorption losses in the phycobilisome‐based LSC were ≈50% lower than a reference sample based on partially decoupled phycobilisomes, indicating the effectiveness of FRET systems in tackling this loss mechanism.

#### 3.3.3 Hybrid FRET Systems

Organic lumophores can also be grafted to the surface of inorganic lumophore particles to form hybrid FRET systems. Inorganic lumophores, such as quantum dots (QDs),^[^ ^64^ ^]^ perovskite nanoparticles,^[^ ^65^ ^]^ and lanthanide complexes^[^ ^66^ ^]^ form another sub‐class of luminescent materials that are widely used in LSCs. QDs are typically semiconductor nanoparticles with diameters of 1–100 nm^[^ ^67^ ^]^ and are particularly popular for LSCs due to their wide absorption band, tunable spectra, high PLQY, and good photostability.^[^ ^64b^ ^]^ Due to quantum confinement effects,^[^ ^67^, ^68^ ^]^ the photophysical behavior of QDs is similar to that of organic lumophores. Reabsorption and subsequent energy losses are a major problem for LSCs made of conventional QDs, due to the large overlap between their absorption and emission spectra.^[^ ^28^, ^69^ ^]^ This problem has been overcome with the advent of so‐called Stokes shift engineering using core‐shell type structures, where a large shell prepared from a wide bandgap semiconductor is grown on a relatively small core prepared from a narrower bandgap semiconductor (e.g., CdSe, InP).^[^ ^23^, ^28^ ^]^ In these materials, absorption takes place in the shell, while the lower energy emission originates from either the core or interfacial region. Such QDs show zero‐ or negligible reabsorption, as the absorption and emission processes are electronically decoupled, leading to high performance LSCs.^[^ ^28b^ ^]^

**Figure 11:**
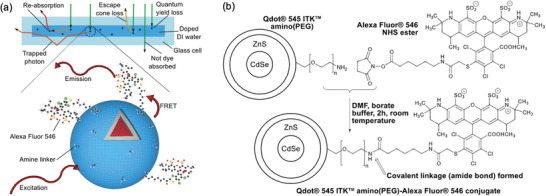
An FRET‐based liquid LSC using surface‐functionalized QD lumophores. a) Schematic representation of the liquid LSC architecture (top) and the FRET process between Qdots 545 and the surface‐anchored AFDye. b) Reaction scheme for covalent grafting of the AFDye to the coating of the QDs. Adapted with permission.^[^ ^16d^ ^]^ Copyright 2017, Elsevier.

Reabsorption losses can also be reduced using an FRET scheme between QDs and organic lumophores.^[^ ^70^ ^]^ In 2016, Tummeltshammer et al. reported an LSC where a small molecular organic emitter, Alexa Fluor 546 dye (AFDye), was attached on the surface of CdSe@ZnS core‐shell QD donor (Qdots 545).^[^ ^16d^ ^]^ The AFDye was covalently anchored via its *N*‐hydroxysuccinimidyl ester moiety to the amino polyethylene glycol amphiphilic coating of the QDs (**Figure** **11**). The optimal *D* *:E* ratio was 1:10, due to the decreased ACQ and reabsorption from the emitter under these conditions, resulting in a moderate FRET efficiency of ≈60%. A series of liquid LSCs was made by injecting aqueous solutions of the QDs into a glass cell (20 × 20 mm^2^), with application of a diffuse reflector to the bottom of the cell. The experimental PCE of the LSC with the grafted donor–emitter QDs was 2.87%, 28% higher than the QD‐only LSC and 10% higher than the LSC with the physically mixed *D*–*E* system at the same concentration ratio. The authors suggest that the PCE enhancement observed for the grafted *D*–*E* QD LSCs should be greater for larger devices, as the associated reabsorption losses would be more pronounced in the longer optical path for the less efficient FRET systems.

### 3.4 The Role of the Host in FRET‐LSCs

While the properties of the donor–emitter pair, that is, spectral overlap, PLQY and separation, are the primary factors determining the FRET efficiency, the host and/or lightguide material and the architecture can also have a significant impact on the final LSC performance. For example, the host material can also be used to control the separation of the *D*–*E* pair, or to eliminate lumophore aggregation, either passively or by design. Moreover, active hosts may also participate in the FRET process, or alternatively can be used to control lumophore alignment to switch FRET on/off using an external stimulus. Examples of these strategies are discussed in more detail in the following sections.

#### 3.4.1 Photoactive Host Systems

**Figure 12:**
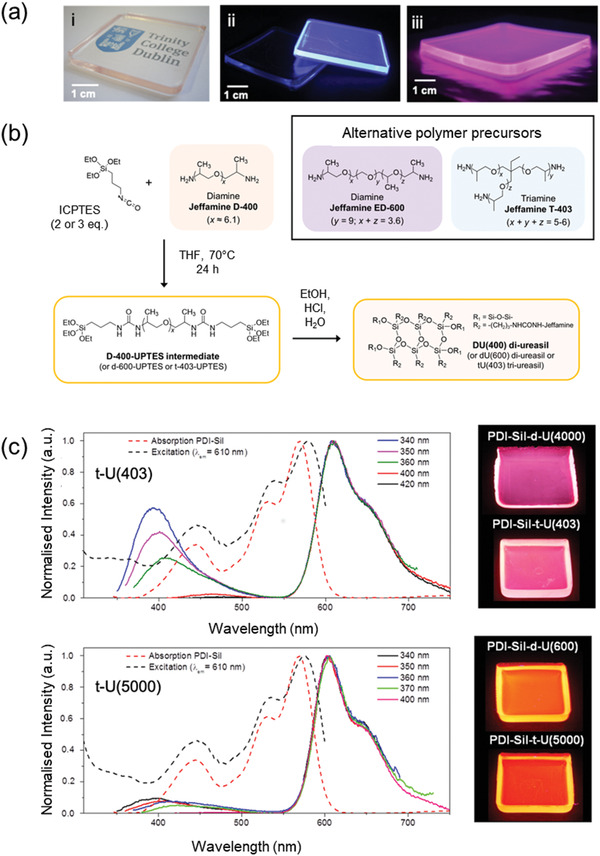
Photoactive lightguides based on ureasils can participate in FRET processes in LSCs. a) Photographs of representative ureasil samples under i) visible light and UV illumination for ii) pure and single lumophore doped ureasils and iii) a two‐lumophore FRET sample. Adapted with permission. Adapted with permission.^[^ ^73b^ ^]^ Copyright 2017, Royal Society of Chemistry. b) Representative synthetic route to a (di)‐ureasil hybrid. The structure can be varied by changing the polymer precursor. Adapted with permission.^[^ ^71^ ^]^ Copyright 2020, SPIE. c) Absorption/excitation and emission spectra of PDI‐Sil doped tri‐ureasils. The efficiency of energy transfer from the ureasil host (≈370–500 nm) to the PDI‐Sil emitter (≈550–750 nm) can be controlled by varying the molecular weight of the polymer backbone (e.g., from 403 to 5000 g mol^−1^), as demonstrated by the degree of quenching of the ureasil emission and the observed total sample emission (see photographs on right). Adapted with permission.^[^^74d]^ Copyright 2016, Royal Society of Chemistry.

In the examples described so far, the FRET pair has typically been dispersed in a thin film or bulk conventional polymer, often PMMA or its derivatives. In this design, the role of the polymer is simply to host the lumophores and guide the emitted light. Recently, a family of organic–inorganic hybrid polymers known as ureasils,^[^ ^71^ ^]^ have been demonstrated to not only satisfy these basic host requirements, but also to provide additional benefits due to their intrinsic photoluminescence (**Figure** **12**a). While ureasils are optically transparent under visible light, they exhibit blue photoluminescence under UV irradiation (365 nm).^[^ ^71^, ^72^ ^]^ The general ureasil structure comprises a siliceous backbone which is chemically grafted via urea bridges to poly(ethylene oxide)/poly(propylene oxide) chains (Figure 12b). However, the mechanical and optical properties can be easily tuned by changing the branching, molecular weight, or chemical composition of the polymer backbone.^[^ ^71^ ^]^ The PL arises from a combination of two radiative processes, namely photoinduced proton transfer at the urea linkages and electron–hole recombination of localized oxygen defects in the siliceous nanodomains.^[^ ^72a^ ^]^ As such, the PL spectrum shows strong excitation wavelength dependence, due to the relative absorptivity of the two species at a particular spectral position.^[^ ^71^ ^]^ The PLQY of ureasils varies approximately from 4% to 16% depending on the structure of the polymer precursor and the conditions used for the synthesis.^[^ ^71^, ^73^ ^]^

Due to their inherent photoluminescence, ureasils have been explored as active hosts for inorganic ions/complexes,^[^ ^75^ ^]^ conjugated polymers,^[^ ^48^, ^72^, ^73^ ^]^ and molecular dyes^[^ ^74^ ^]^ in which the excitation wavelength dependence of the ureasil is exploited to modulate energy transfer between the host and dopant or expand the light‐harvesting window. In 2016, Kaniyoor et al. reported a planar bulk LSC based on a di‐ureasil lightguide incorporating LR305 as the lumophore.^[^ ^74b^ ^]^ Overlap between the di‐ureasil emission and LR305 absorption bands resulted in energy transfer from the host to the lumophore, which effectively extended the absorption window of the final LSC device. This, combined with the near‐unity PLQY of LR305, yielded an optical power efficiency (*η*_opt_, the ratio of summed edge output light power to the incident light power on the surface of the LSC) of 14.5% under solar simulated light (300–800 nm) at an optimized lumophore concentration of 0.005 wt%, demonstrating that ureasil lightguides performed just as well as standard PMMA hosts.^[^ ^74b^ ^]^ However, LR305 showed a tendency to aggregate in the di‐ureasil host at concentrations > 0.05 wt%, which resulted in the slight reduction in its PLQY and red‐shift in its emission spectrum. Meazzini et al. attempted to tackle this issue by covalently grafting the lumophores to the ureasil matrix to prevent aggregation.^[^ ^74d^ ^]^ This was achieved by decorating a perylene diimide with alkoxysilane groups (PDI‐Sil, Figure 5b), which allowed selective grafting of the lumophore to the siliceous domains of the ureasil hosts. This strategy enabled retention of the high PLQY in the solid‐state (76–87% on direct PDI‐Sil excitation). Moreover, the spatial distribution of the PDI‐Sil molecules in the hybrid matrix could be altered by tuning the branching or molecular weight of the polymer backbone in the ureasil (Figure 12c). This led to a variation in the FRET efficiency and thus to a tunable emission color in the final composite materials.^[^ ^74d^ ^]^ This approach was later extended to design a FRET‐LSC (4 × 4 × 0.3 cm^3^) incorporating a blue‐emitting conjugated polyelectrolyte donor and an LR305 acceptor (Figure 12a), which exhibited an *η*_opt_ of 13% on a scattering background under solar simulated light.^[^ ^73b^ ^]^

Ureasil hosts have also been used to develop near‐infrared (NIR) emitting thin‐film LSCs based on FRET from the host to the embedded lumophore, silicon 2,3‐naphthalocyanine bis(trihexylsilyloxide) (SiNc), cast on a glass substrate.^[^ ^74e^ ^]^ Despite the low PLQY of SiNc, the resulting LSC device still achieved a *η*_opt_ of 1.5% under AM 1.5 G irradiation, attributed to negligible reabsorption losses as a result of the large Stokes shift of the SiNc lumophore.^[^ ^74e^ ^]^ The same group subsequently adopted chlorophyll as an LSC lumophore in di‐ and tri‐ureasil hosts.^[^ ^74a^ ^]^ While energy transfer from both hosts to the lumophore was confirmed, as indicated by the quenching of ureasil emission and the reduction in the corresponding fluorescence lifetime with increasing doping concentration, the process was more efficient between the tri‐ureasil and chlorophyll. This was attributed to the strong interaction between the chlorophyll molecules and the amide‐based emitting centers of the tri‐ureasil host. Moderate *η*_opt_ and PCE values of 3.7% and 0.1% (AM 1.5 G irradiation), were obtained, respectively, for the optimized chlorophyll‐di‐ureasil hybrid sample, demonstrating the potential of using chlorophyll as a red/NIR bio‐emitter.^[^ ^74a^ ^]^

Due to easy processability and mechanical flexibility, ureasils can be fabricated into a variety of geometries. In 2015, Correia et al. reported cylindrical LSCs based on PMMA fibers coated with a layer of Eu^3+^‐doped di‐ureasil.^[^ ^14c^ ^]^ Eu^3+^ ions were sensitized by TTA (2‐thenoyltrifluoroacetonate) ligands and features of both the ureasil host (≈280 nm) and the TTA ligands (≈330–370 nm) were observed in the excitation spectrum detected in the ^5^D_0_→^7^F_2_ transition of Eu^3+^ ion. This indicated the presence of energy transfer from both the ureasil and TTA ligands to the Eu^3+^ ion centers, effectively extending the solar‐absorption region of the device. It is worth noting that the FRET mechanism (dipole–dipole interactions) is less effective in lanthanide‐based systems, due to the parity‐forbidden nature of f–f transitions. As such, the electron exchange (Dexter) mechanism operates in this system, due to the short (<0.5 nm) distance between the Eu^3+^ center and the TTA ligands.^[^ ^76^ ^]^ The final LSC device exhibited excellent *η*_opt_ and PCE of 20.7% and 2.5%, respectively (measured under AM 1.5 G irradiation in 300–380 nm region).^[^ ^14c^ ^]^ They subsequently investigated the scale‐up of cylindrical LSCs, where the light‐harvesting component was based on either a di‐ or tri‐ureasil host doped with a Eu(TTA)_3_·H_2_O complex or rhodamine‐6G (Rh). The typical emission of the undoped ureasil host was absent in the emission spectra of both the Eu^3+^‐ and Rh‐ureasil composites, indicating effective energy transfer from the ureasil host to the embedded lumophores. A high flux gain, *F* of 12.3 was obtained for the optimized cylindrical hollow‐core LSC system comprising Eu(TTA)_3_·H_2_O complex in a t‐(5000) ureasil, which was among the highest *F* values reported for LSCs at this time.^[^ ^14b^ ^]^

#### 3.4.2 Host‐Driven Lumophore Alignment

**Figure 13:**
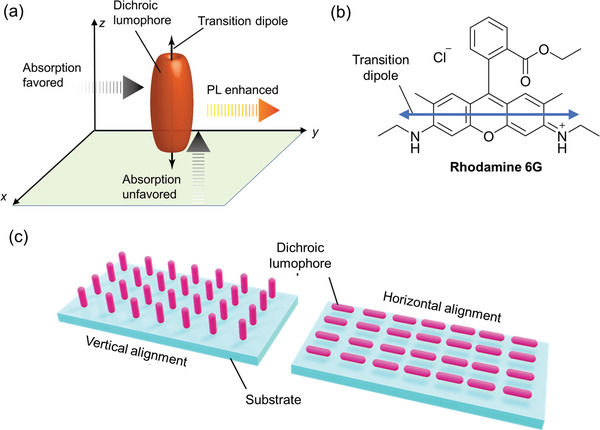
The intensity of absorption and emission depends on lumophore alignment. a) In planar organic lumophores, the transition dipole moment (TDM) is located along the long axis of the molecule. Absorption is favored when the electric field vector of the incident photon is parallel to the TDM (i.e., perpendicular to the direction of travel), and unflavored in the orthogonal axis. Similarly, photoluminescence is enhanced under the same conditions. Adapted with permission.^[^ ^50^ ^]^ Copyright 2019, American Chemical Society. b) An example of a dichroic molecule, Rhodamine 6G, showing the orientation of the transition dipole moment. c) Lumophore alignment in an LSC will affect the absorption and emission properties. In the vertically aligned LSC, the TDM of the dichroic lumophore is perpendicular to the substrate surface, leading to reduced absorption. In the horizontal alignment, the TDM is parallel to the substrate surface and, quite often, two opposite edges of the LSC, which can lead to reabsorption losses.

In conventional LSCs, the lumophores are randomly oriented in/on the lightguide, leading to isotropic absorption and emission properties. However, some host materials can be used to selectively orientate or align lumophores in LSCs, which can enhance their concentration factor.^[^ ^20^, ^78^ ^]^ For most organic lumophores with asymmetrical molecular structures, absorption and emission are greatest when the transition dipole moment is parallel to the electric field vector of the incoming/outgoing photon (**Figure** **13**a,b), the so‐called dichroism effect.^[^ ^78^ ^]^ Two basic extremes of lumophore alignment can be envisaged for LSCs: horizontal and vertical (Figure 13c).^[^ ^20^, ^78^ ^]^ For example, dichroic lumophores can be aligned such that their final emission is focused to the desired direction (e.g., one or more edge of the lightguide). However, the anisotropic absorbance of an aligned lumophore system can be unfavorable, leading to reduced incident light absorption for vertical alignment and increased reabsorption in the focused emission direction for both types of alignment.^[^ ^20^, ^78^ ^]^ A FRET approach can be used to overcome both problems.

In the typical horizontal alignment, the excited‐state dipole moments of the lumophores are aligned parallel to two opposite edges of the LSC (Figure 13b). As the emission is confined to two parallel edges instead of four, this strategy helps to concentrate more light onto a smaller area, similar to the effect of increasing the geometric gain of the LSC. Mulder et al. used this approach to design a horizontally‐aligned LSC based on a FRET pair of Coumarin 6 as the donor and 4‐(dicyanomethylene)‐2‐methyl‐6‐(4‐dimethylaminostyryl)‐4*H*‐pyran (DCM) as the emitter.^[^ ^77b^ ^]^ To create the horizontally‐aligned profile, a thin layer of polyimide acid was first spin‐coated on a glass substrate, and manually rubbed with a velvet cloth in the desired direction of the alignment. This is a commonly used approach to produce horizontally‐aligned polymeric films.^[^ ^77a^ ^]^ A polymerizable nematic liquid crystal host material, (LC242, BASF) was blended with both lumophores and spin‐coated on top of the pre‐aligned polyimide acid films. The sample was then exposed to UV light (350 nm) for 3 min to trigger polymerization of the liquid crystal, giving a solidified lightguide with a horizontally‐aligned lumophore layer on top. The alignment was confirmed by the polarized absorption spectrum of the Coumarin 6/DCM blend sample, where the polarized absorption was ≈90% parallel to the alignment direction, but only ≈60% in the perpendicular direction. The emission of the sample was dominated by DCM, confirming an efficient FRET process in the horizontally aligned system. The emission of the Coumarin 6‐only sample to the parallel edges was twice as intense as the emission at the perpendicular edges. However, the *η*_ext_ (laser excitation at 465 nm) measured from the parallel edges decreased faster than from the perpendicular edges with increasing *G*, since reabsorption was also enhanced due to the preferential lumophore alignment in this direction.

Another example of the horizontal alignment of an FRET lumophore pair was reported in 2020 by Timmermans et al.^[^ ^79^ ^]^ In this work, a reversible liquid crystal network (LCN) system was first prepared, in which some hydrogen bonds in the network could be opened in aqueous basic conditions (0.1 m KOH) to create a nanoporous structure. The donor lumophore, a coumarin derivative DFSB‐K160, was aligned by the LCN, while the emitter lumophore, Rhodamine B, was doped into the nanopores after alkaline treatment. This approach led to improved distribution of the emitters in the nanopores, resulting in a 70% FRET efficiency, with potential for LSC applications.

In vertically‐aligned LSCs, the lumophores are arranged such that their transition dipole moments are aligned perpendicular to the top and bottom surfaces of the lightguide. This arrangement effectively confines photons emitted parallel to the lightguide surface, leading to reduced escape‐cone losses.^[^ ^77c^ ^]^ The emission of vertically‐aligned LSCs remains isotropic in the horizontal direction, in contrast to horizontally‐aligned LSCs. However, as the vertically aligned dipoles are also parallel to the incident light of the LSCs, absorption is weak and transmittances losses can increase significantly. To overcome this, front and rear diffuser layers can be used to scatter the incident light to improve the absorption of vertically‐aligned LSCs.^[^ ^20^, ^81^ ^]^ However, it is not an ideal solution, as the diffuser layers may also introduce more reflection losses at the top surface of the device.

**Figure 14:**
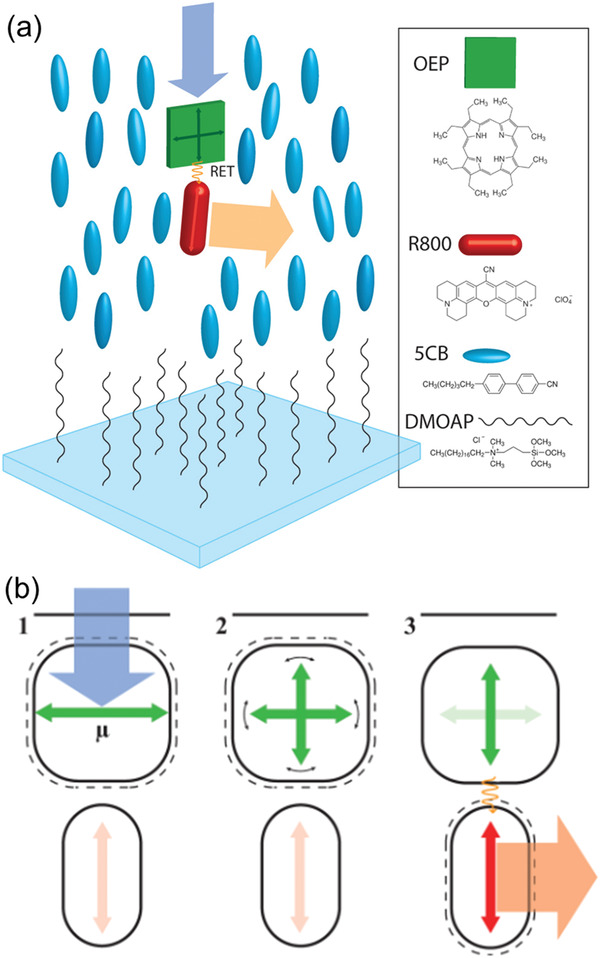
Poor light absorption in vertically‐aligned LSCs can be overcome using a non‐dichroic donor with a dichroic emitter. a) Schematic diagram of the OEP (donor)/R800 (emitter) FRET pair vertically aligned by the liquid crystal matrix, and b) the decoherence of the degenerated transition dipole pair in OEP. One dipole absorbs the incident light and the second transfers energy to the emitter via FRET. Adapted with permission.^[^ ^81^ ^]^ Copyright 2013, American Chemical Society.

FRET systems are used more often in vertically aligned LSCs to address this issue of increasing transmittance loss. The biggest contradiction in vertically‐aligned LSCs is that the transition dipoles of the lumophores are perpendicular to the favored orientation of light‐harvesting. A FRET system effectively solves this problem by distributing the roles of absorption and emission to the donor and emitter, respectively. To date, there are two approaches to apply this strategy in vertically‐aligned LSCs. One approach is to pair a non‐dichroic donor with a dichroic emitter, as reported in 2013 by MacQueen et al.^[^ ^81^ ^]^ In this work, the donor, octaethyl porphyrin (OEP), and the emitter, rhodamine 800 (R800), were vertically‐aligned in a liquid crystal matrix, 4‐cyano‐4‐n‐pentylbiphen (5CB) (**Figure** **14**a). OEP has two degenerate transition dipoles which are orthogonal to each other (Figure 14b). Excitation to one particular dipole can be rapidly decohered to both transition dipoles with equal probability. Under vertical alignment, OEP can absorb light through its dipole that is aligned parallel with the lightguide and transfer the energy to R800 through its perpendicular dipole (Figure 14b). Both the absorption of the lightguide and the FRET efficiency are improved using this approach.

Another example was reported by Tummeltshammer et al. through Monte‐Carlo simulation of an FRET system based on a *D*–*E* combination of PbS QDs grafted to C6 vertically aligned in the lightguide.^[^ ^82^ ^]^ The absorption of QDs is not influenced by the vertical alignment, due to their non‐dichroic nature. The energy from the absorbed photon could be transferred to the vertically‐aligned C6 moieties via FRET and emitted parallel to the lightguide surface. The simulated *η*_opt_ of such an LSC (1000 × 1000 × 4 mm^3^) in perfect vertical alignment is 27.7%, much higher than 1.1% of the QD‐only system. However, this idea has not been realized in practice to date, partially because of the difficulties in dispersing QDs in liquid crystal matrices.

The second approach to minimize transmittance losses in vertically‐aligned LSCs is to use a selectively aligned FRET system, in which the emitter molecules are vertically aligned, and the donor is randomly oriented. One such system was reported by Pieper et al. in 2018,^[^ ^83^ ^]^ where Coumarin 1 and Coumarin 6 were blended in a matrix of polyvinyl alcohol (PVA) as the donor and emitter, respectively, and deposited as a membrane on a glass substrate via the drop‐casting method. The lumophore‐doped PVA membrane was mechanically stretched to 400% in length, leading to the alignment of Coumarin 6 in the stretching direction, while leaving Coumarin 1 in random orientations. The paper suggested that this approach could be adopted to produce LSCs comprising vertical assemblies of stretched‐aligned PVA layers, although such devices were not practically demonstrated in this study.

**Figure 15:**
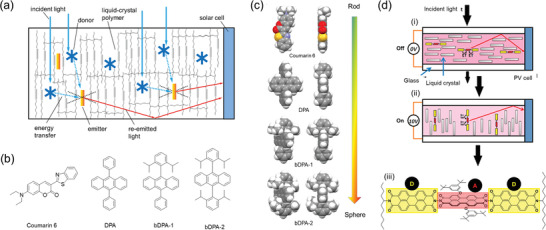
Tunable alignment of molecular lumophores in liquid crystal matrices. a) A FRET‐LSC based on rod‐shaped emitters aligned vertically the liquid crystal matrix, while the spherical donors remained in random orientation to absorb the incident light. b) Chemical structures and c) molecular shapes of the emitter and donors. Adapted with permission.^[^ ^50^ ^]^ Copyright 2019, American Chemical Society. d) A switchable LSC in which the lumophore alignment can be switched between i) absorbing and ii) transparent modes upon application of an electric field. iii) Chemical structure of the donor–emitter‐donor intramolecular FRET triad used as the lumophore in this LSC. Adapted with permission.^[^ ^78^ ^]^ Copyright 2014, American Chemical Society.

In 2019, Zhang et al. demonstrated an alternative method for lumophore alignment of FRET pairs which exploited how the molecular “shape” of donor or emitter molecules governed the interaction with the host material.^[^ ^50^ ^]^ A UV‐curable nematic liquid‐crystal polymer, UCL018, was used as the host matrix, which was aligned vertically on glass substrates via self‐assembly (**Figure** **15**a). Coumarin 6 was used as the emitter, due to its strong interaction with the liquid crystal host because of its “rod‐like” shape. To achieve selective alignment, the molecular “shapes” of the donors (diphenyl anthracene (DPA) and two derivatives, bDPA‐1 and bDPA‐2) were specifically designed to vary from planar to spherical, to vary the interaction with UCL018 (Figure 15b,c). As expected, the planar DPA could be vertically‐aligned by the matrix. However, the spherical bDPA‐2 precipitated due to the poor compatibility with the matrix, while the ellipsoidal bDPA‐1 showed an intermediate interaction with UCL018, in that it neither precipitated nor aligned. A mixture of *D*–*E* lumophores and UCL018 were spin‐coated on glass substrates and immediately cured by UV light to lock‐in the vertical alignment. The absorptivity of Coumarin 6 in UCL018 was 60% lower in comparison with the reference sample in PMMA, indicating efficient vertical alignment in the liquid crystal film. However, the absorptivity of bDPA‐1 only decreased by <10%, suggesting that the ellipsoidal bDPA‐1 molecules were not vertically aligned. The optimized FRET pair of bDPA‐1 (50 mm) and Coumarin 6 (8 mm) had a PLQY of ≈98%, indicating efficient donor‐to‐acceptor energy transfer. The trapping efficiency of the resulting vertically‐aligned LSC was 79%, which was comparable to other vertically‐aligned LSCs,^[^ ^20b^ ^]^ but with a much lower penalty to the transmittance loss.

Other studies have exploited the use of an external stimulus such as electric fields to control the alignment of lumophore in liquid crystalline hosts.^[^ ^84^ ^]^ This approach has been used in smart windows^[^ ^85^ ^]^ and recently applied to switchable LSCs.^[^ ^86^ ^]^ ter Schiphorst et al. reported a *D*–*E*–*D* intramolecular FRET triad based on two types of PDI units (Figure 15d).^[^ ^78^ ^]^ The excitation spectrum of the PDI triad monitored at 630 nm, where the donor PDI moiety shows no fluorescence, is identical to its absorption spectrum, suggesting efficient FRET. The PDI triad was incorporated into a commercial liquid crystal matrix (MLC‐6284‐000, Merck Licristal) and sandwiched between two pieces of ITO‐coated glass. The LSC could be switched from the absorbing to the transparent mode by applying a voltage of 10 V across the device. In the absorbing mode, all triad molecules were aligned in the direction parallel to the lightguide surface, while in the transparent mode, they were aligned perpendicular to the surface (Figure 15d). The ratio between the integrated absorbance of the absorbing mode and that of the transparent mode was 2.83 for the LSC based on the FRET triad. In comparison, the ratio of absorbance was only 1.87 for the reference sample using a simple mixture of PDI monomers as lumophores. The ratio for the edge emission output was 2.02 for the triad LSC and 1.65 for the reference LSC. The results suggest that alignment induces more anisotropy in both the absorption and emission of the rigid triad PDI than the simple mixture of PDIs.

The alignment of some liquid crystal matrices is not only responsive to electric fields but also to variations in the local temperature. This has been exploited to fabricate a FRET‐based temperature‐responsive LSC, based on a coumarin derivative as the donor and PDI as the emitter, which were blended in a liquid crystal host mixture, E7 (Merck KGaA), before loading into an alignment cell.^[^ ^87^ ^]^ Similar to the aforementioned switchable LSCs^[^ ^78^ ^]^ the lumophores in this device can be vertically aligned by applying a voltage bias of 10 V across the alignment cell. However, the phase transition of the liquid crystal matrix was also responsive to the temperature of the surrounding environment, which affected both the FRET behavior and the alignment profile of the device. At room temperature, the matrix was in the crystalline phase. In this mode, the emitter precipitated as aggregates, but the donor remained dissolved in the aligned matrix, leading to emission predominantly from the donor. When the temperature was increased to 70 ℃, the matrix became liquid. The emitter aggregates then dissolved in the matrix, which triggered the FRET process, causing the PL spectrum of the device to be dominated by the emission of the emitter. The estimated FRET efficiency was 54% in the hot state. A LSC was made of the FRET pair in a 5 × 5 cm^2^ area confined within the alignment cell gap of 20 µm, giving a *η*_opt_ of 2.4% and 3.2% in the cold and heated states, respectively. This work provided an approach to tune the photophysical properties of FRET‐LSCs via both thermal and electric stimuli.

## 4 Guidelines for Designing FRET‐LSCs

A review of the recent literature has shown that FRET systems have proven to be an efficient strategy for reducing reabsorption, extending the absorption window, and improving the PLQY of LSCs. The key features the FRET enhanced LSCs discussed in this review are summarized in **Table** **1**. Moreover, we have established that the efficiency of FRET systems for LSCs can be boosted through consideration of four key criteria, namely i) optimization of the spectral overlap, ii) increasing the PLQY of both the donor and emitter lumophores, iii) minimizing the intermolecular distance between FRET pairs, and iv) manipulating the interactions between the lumophores and host to control lumophore placement and alignment and to enhance the optical properties.

**Table 1:** Summary of the FRET lumophore systems and LSCs reviewed in this work

	Donor	Emitter	Distribution method	FRET efficiency	PLQY	Performance of LSC	Size	Ref
1	PEAs (P1 and P2)	LR305	PMMA spin‐coat on glass	80–88%	79%	*η*_ext_= 40% at 405 nm	17.5 × 17.5 × 1.5 mm^3^	[34]
2	BTBBT	PE‐Pery LR305	PMMA on glass	–	≈100%	*η*_ext_= 8.6% in AM 1.5 G	50 × 50 × 3 mm^3^_,_ 25 µm of PMMA	[35]
3	PDI	LR305	PMMA on glass	–	82%	PCE = 0.46% in 1 sun	5 × 5 × 0.1 cm^3^	[39]
4	bPDI‐4	LR305	PMMA on glass	Fully quenched	90%	*η*_int_= 56% at 490 nm (simulated)	480 × 480 × 1 mm^3^, *G* = 120 (simulated)	[19a]
5	bPDI‐3	LR305	PMMA on PMMA	>95%	97.6%	*F* = 7.4, PCE = 2.59% in AM 1.5 G	200 × 200 × 1 mm^3^, *G* = 50	[16c]
6	–	TPE	PMMA on quartz	–	49%	*η*_ext_= 13.2% at 320 nm	10 × 10 × 1 mm^3^	[44]
7	DPATPAN	DCJTB	PMMA on glass	Fully quenched	92%	*η*_int_= 58.2% at 500 nm	1.2 × 1.2 × 0.1 cm^3^	[47]
8	DPATPAN	PITBT‐TPE	PMMA on glass	Fully quenched	>90%	*F =* 11.6 at 390 nm (simulated)	*G* = 50 (simulated)	[25b]
9	p‐O‐TPE	PDI‐Sil	Bulk ureasil	–	45%	*η*_int_ = 20% in AM 1.5 G	4.5 × 4.5 × 0.3 cm^3^	[48]
10	Rubrene	DCJTB	Alq_3_ on glass	–	–	Estimated PCE = 13.8% in AM 1.5 G (*G* = 45)	100 × 100 × 1 mm^3^	[16b]
11	NDI, PDI	LR305	PMMA particles doped in LMA‐EGDM copolymer	Fully quenched	95%	Simulated *F* = 12 in AM 1.5 G	100 cm^2^ (simulated)	[51]
12	DPH, DBT	DBTT	DCA on quartz	Fully quenched	67%	–	–	[52]
13	Anthracene	Tetracene	Cocrystal in PMMA	Nearly fully quenched	–	*η*_ext_= 23.72% in AM 1.5 G	25 × 25 × 1.8 mm^3^	[42]
14	Perovskite nanoplatelets	Perovskite nanoplatelets	PMMA on glass	–	75%	*η*_ext_= 0.87% in AM 1.5 G	10 × 10 × 0.2 cm^3^	[45]
15	PMI	PDI (A1, A2)	Glass	>99.9%	80	*F* = 4.88 in AM 1.5 G (simulated)	*G* = 132 (simulated)	[57, 58]
16	PDI (SM)	PDI (AM)	TBA pendant copolymers	Fully quenched	–	–	–	[56]
17	PDI (SM)	PDI (AM)	TBA, MMA pendant copolymers	Fully quenched	>80%	–	–	[61]
18	BODIPY subunits	BODIPY subunits	BODIPY dendrimer	>90%	32%	–	–	[60]
19	Oligofluorene	BODIPY	Dendrimer	Fully quenched	66–75%	*η*_ext_= 2.44% in AM 1.5 G	10 × 10 × 0.3 cm^3^	[62]
20	Phycoerythrin, phycocyanin	Allophycocyanin	Bound with phycobilisome in polyacrylamide hydrogel	Mostly quenched	<50%	*η*_ext_= 12.5% at 560 nm	22 × 22 × 0.5 mm^3^	[63]
21	CdSe@ZnS QD	Alexa Fluor 546	Solution	≈60%	–	PCE = 2.87% in AM1.5	20 × 20 mm^2^	[16d]
22	Ureasil	LR305	Bulk ureasil	–	–	*η*_opt_ = 14.5% in simulated sunlight (300–800 nm)	4 × 4 × 0.3 cm^3^	[74b]
23	Ureasil	PDI‐Sil	Bulk ureasil	–	76–87%	*–*	–	[74d]
24	Polyelectrolyte	LR305	Copolymer in bulk ureasil	–	65.1%	*η*_opt_ = 13% in AM 1.5 G	4 × 4 × 0.3 cm^3^	[73b]
25	Ureasil	SiNc	Ureasil film on glass substrate	–	16%	*η*_opt_ = 1.5% under AM 1.5 G	7.6 × 2.6 ×0.1 cm^3^	[74e]
26	Ureasil	Chlorophyll	Ureasil film on glass substrate	–	16%	*η*_opt_ = 3.7% in AM 1.5 G	7.6 × 2.6 ×0.1 cm^3^	[74a]
27	Ureasil/TTA	Eu^3+^ (Dexter)	Ureasil coated on PMMA fiber	–	61%	*η*_opt_ = 20.7% in 300–380 nm of AM 1.5 G	Cylinder *L* = 5 cm *D* = 0.3 cm	[14c]
28	Ureasil/TTA	Eu^3+^ (Dexter)	Hollow‐core PMMA/ureasil fiber	–	85%	*F =* 12.3 in 300–380 nm of AM 1.5 G	Cylinder *L* = 1.5 m *d*_out_ = 1.6 mm *d*_in_ = 0.84 mm	[14b]
29	Coumarin 6	DCM	Liquid crystal (LC242) on pre‐aligned polyimide acid films	Fully quenched	60%	*η*_int_ = 25% at single wavelength *η*_ext_ ≈ 28%at 465 nm on the perpendicular dipole	2 × 2 cm^2^ in *η*_int_ measurement 7.6 × 9.5 cm^2^ in *η*_ext_ measurement	[77b]
30	DFSB‐K160	Rhodamine B	Liquid crystal network	70%	–	*–*	–	[79]
31	OEP	R800	Liquid crystal 5CB	–	–	*–*	–	[81]
32	PbS QD	Coumarin 6	QD grafted to C6 Vertically aligned	–	–	*η*_opt_= 27.7% in AM 1.5 G (simulated)	1000 × 1000 × 4 mm^3^ (simulated)	[82]
33	Coumarin 1	Coumarin 6	PVA	–	83%	–	–	[83]
34	bDPA‐1	Coumarin 6	Liquid crystal UCL018	83%	98%	*η*_ext_ = 10.8% at 375 nm	1.25 × 1.25 × 0.1 cm^3^	[50]
35	PDI (donor)	PDI (emitter)	PDI‐triad MLC‐6284‐000	Fully quenched	–	–	–	[78]
36	Coumarin derivative	C12‐PBI	Liquid crystal E7	54%	–	*η*_opt_ = 3.2% in AM 1.5 G (hot state)	5 × 5 cm^2^ 20 µm cell gap	[87]

Based on these criteria, we propose the following step‐by‐step guidelines should be followed when designing a new FRET system for LSCs to maximize the efficiency of the end device:

- Identify the desired spectral operating window of the LSCs that will both maximize light absorption that is, good overlap with the solar spectrum or lamp profile (for indoor applications) and emission overlap with the spectral response of the attached PV cell (for LSC‐PV applications).
- Select a *D*–*E* pair whose spectral features complement the desired operating window—namely the donor absorption spectrum should overlap significantly with the incident light and the PL spectrum of the emitter should meet the requirements of the desired spectral output. Ideally, the absorption from the emitter in the incident spectral region should be minimized to further reduce reabsorption.
- Determine the critical FRET radius (*R*_0_ see Equation (7)) to ensure the lumophore pair is suitable for FRET. Typical values for *R*_0_ are 2–6 nm for efficient systems.^[^ ^32^ ^]^
- Identify an approach to ensure that the intermolecular distance between the *D*–*E* pairs falls within *R*_0_, for example, by localizing the lumophore pair in discrete aggregates or controlling the volume of lumophore dispersion.
- Optimize the PLQY for both the donor and emitter lumophores by considering strategies to prevent undesirable aggregation (ACQ) or promote favorable aggregation (AIE). The observed PLQY of the donor and emitter should be >90% for an efficient system.
- Optimize the concentration and ratio of the donor and emitters to ensure sufficient quenching of the donor's emission and thus reduce further reabsorption. An observed FRET efficiency > 90% is ideal. The local concentration of the emitter should be >5 mm to ensure sufficient quenching of the donor.
- Consider the use of responsive or active host, substrate, or additive materials that may further improve the performance of FRET‐LSCs or provide additional functionality.

## 5 Outlook for FRET‐LSCs

Although the concept of FRET‐LSCs was developed nearly half a century ago, they remain an active area of research for two main reasons. First, LSCs in general have still not penetrated the commercial market as their performance particularly in LSC‐PV configurations remains unsatisfactory upon scale‐up due to intrinsic optical losses. However, as we have seen, FRET provides a potential solution to the primary scale‐up loss of reabsorption. Moreover, alternative LSC configurations and applications beyond LSC‐PV are increasingly emerging,^[^ ^6a^ ^]^ in which the tunable spectral features offered by FRET systems may be beneficial. Second, there has been a revolution in the design of luminescent materials in recent years, such that new potential candidates for FRET systems including QDs, perovskite nanocrystals or AIEgens, worthy of further investigation for LSCs are frequently being identified. Herein, we propose some future directions and knowledge gaps that may help to further develop FRET‐enhanced LSCs.

Conventional LSCs are normally produced by directly dispersing the lumophores into a host matrix, either as a thin film which is then cast onto a lightguide substrate, or in a bulk configuration where the host itself is the lightguide. As discussed, the FRET efficiency is highly dependent on the intermolecular distance between the donor–emitter pair in the host material. Preliminary reports on the use of confined FRET systems, where the lumophore pair are co‐localized in a nanoparticle structure, either through co‐crystallization or through a core‐shell type structure, have shown this to be a promising strategy worth of further investigation. However, it is crucial that these photoactive nanoparticles are homogeneously distributed within the host material to maximize absorption and minimize undesirable non‐radiative loss pathways caused by aggregation. Strategies to control the placement and/or dispersion of nanoparticles in typical host polymers, for example through selective grafting to surface ligands, should also be explored.

Lumophore selection is also important. While classical organic lumophores such as coumarin and perylene diimide derivatives have been explored frequently for FRET systems, there are still relatively few examples based on inorganic lumophores, such as QDs and perovskite nanocrystals. Inorganic lumophores offer the advantages of tunable optical properties, broadband absorption spectra in the visible range, and excellent photostability in ambient conditions. A common challenge is that the optical properties are highly dependent on the size and morphology of the nanostructure; while reproducible synthetic protocols for QDs are well‐established (e.g., hot injection), similar procedures for perovskite nanocrystals and carbon nanodots are still under investigation. Once this challenge is surmounted, it should be possible to make a more critical assessment of the suitability of such lumophores for FRET‐LSCs. Reabsorption losses can also be a problem for inorganic nanostructures due to the small Stokes shift. As discussed, this has been successfully overcome for conventional QDs through nanostructural engineering of the nanoparticle composition^[^ ^23^, ^28^ ^]^ and it is reasonable to envisage that comparable solutions may eventually be found for perovskite nanocrystals and single element QDs (e.g., C or Si). In the meantime, hybrid FRET systems which combine organic and inorganic lumophores, either as discrete nanoparticles or through blends, are an attractive alternative approach.

Aligned FRET systems may open up new possibilities for LSCs that have yet to be fully explored. In single lumophore horizontally‐aligned systems, the absorption/emission transition dipoles are aligned parallel to two edges of the LSCs (for a conventional square/rectangular geometry), leading to increased reabsorption in the same direction as the directed light path. The use of an FRET pair to decouple absorption and emission events could potentially reduce reabsorption, while further improving the FRET efficiency through alignment. However, to date, there is only one report of a horizontally‐aligned FRET‐LSC,^[^ ^77b^ ^]^ indicating there is plenty of scope for evaluating this concept further.

For most vertically‐aligned FRET pairs in LSCs, the alignment of donor dipoles is not particularly favorable as it both limits the absorption efficiency for light incident on the top surface of the LSCs and restricts the required dipole coupling between the donor and emitter during the FRET process. Due to their spherical shape and isotropic photophysical properties, QDs may avoid alignment from the surrounding host substrate, making them the perfect donor candidates for the vertically‐aligned FRET systems in LSCs. The concept of using QDs in such systems has been proposed,^[^ ^82^ ^]^ but not realized experimentally to date. For both vertically and horizontally aligned systems, the possibility of controlling the alignment on‐demand using an external stimulus is also extremely attractive.

The development of any emerging technology—particularly one directed at renewable energy or sustainability—should clearly give due consideration to environmental impact and end‐of‐life implications. While the detail is beyond the scope of this review, this may include using green chemistry approaches to synthesize key components (lightguide, luminophores etc.), the avoidance of toxic materials (e.g., Cd or Pb‐based QDs, lead halide perovskites), or unethically sourced components. While there have been a few lifecycle analysis studies on LSCs,^[^ ^88^ ^]^ circular approaches to LSC design—particularly in the context of recycling and/or re‐use—are limited.

In conclusion, FRET has been demonstrated as a functional approach to overcome the key loss mechanisms in conventional single lumophore LSCs, namely reabsorption and reduced PLQY. FRET‐LSCs also have the potential to demonstrate enhanced performance by expanding the absorption window or improving spectral overlap with attached PV cells. However, there is still significant scope for improving the efficiency of FRET‐LSCs through the bottom–up design of the photophysical properties of the donor–emitter pair, either through chemical design or use of materials engineering strategies to deliver the required characteristics. Moreover, the strategic development of FRET‐LSCs to deliver the spectral requirements for emerging PV technologies (e.g., perovskite, organic cells) may also bring improved performance.

## Conflict of Interest

The authors declare no conflict of interest.
